# Mitochondrial mechanisms in the pathogenesis of chronic inflammatory musculoskeletal disorders

**DOI:** 10.1186/s13578-024-01259-9

**Published:** 2024-06-08

**Authors:** Kailun Wu, Ju-sheng Shieh, Ling Qin, Jiong Jiong Guo

**Affiliations:** 1 Department of Orthopedics, The Fourth Affiliated Hospital of Soochow University/Suzhou Dushu Lake Hospital, Suzhou, Jiangsu People’s Republic of China; 2 Department of Orthopedics and Sports Medicine, The First Affiliated Hospital of Soochow University, 188 Shizi Street, Suzhou, 215006 People’s Republic of China; 3 Department of Periodontology, School of Dentistry, Tri-Service General Hospital, National Defense Medical Center, Taipei City, 11490 Taiwan; 4 Musculoskeletal Research Laboratory of the Department of Orthopaedics & Traumatology, The Chinese University of Hong Kong, Hong Kong, SAR People’s Republic of China; 5 MOE China-Europe Sports Medicine Belt and Road Joint Laboratory, Soochow University, Suzhou, Jiangsu People’s Republic of China

**Keywords:** Chronic inflammatory musculoskeletal disorders, Mitochondrial dysfunction, Mitochondrial calcium overload, Oxidative stress, Mitochondrial dynamics, Mitophagy, Extracellular vesicles

## Abstract

Chronic inflammatory musculoskeletal disorders characterized by prolonged muscle inflammation, resulting in enduring pain and diminished functionality, pose significant challenges for the patients. Emerging scientific evidence points to mitochondrial malfunction as a pivotal factor contributing to these ailments. Mitochondria play a critical role in powering skeletal muscle activity, but in the context of persistent inflammation, disruptions in their quantity, configuration, and performance have been well-documented. Various disturbances, encompassing alterations in mitochondrial dynamics (such as fission and fusion), calcium regulation, oxidative stress, biogenesis, and the process of mitophagy, are believed to play a central role in the progression of these disorders. Additionally, unfolded protein responses and the accumulation of fatty acids within muscle cells may adversely affect the internal milieu, impairing the equilibrium of mitochondrial functioning. The structural discrepancies between different mitochondrial subsets namely, intramyofibrillar and subsarcolemmal mitochondria likely impact their metabolic capabilities and susceptibility to inflammatory influences. The release of signals from damaged mitochondria is known to incite inflammatory responses. Intriguingly, migrasomes and extracellular vesicles serve as vehicles for intercellular transfer of mitochondria, aiding in the removal of impaired mitochondria and regulation of inflammation. Viral infections have been implicated in inducing stress on mitochondria. Prolonged dysfunction of these vital organelles sustains oxidative harm, metabolic irregularities, and heightened cytokine release, impeding the body’s ability to repair tissues. This review provides a comprehensive analysis of advancements in understanding changes in the intracellular environment, mitochondrial architecture and distribution, biogenesis, dynamics, autophagy, oxidative stress, cytokines associated with mitochondria, vesicular structures, and associated membranes in the context of chronic inflammatory musculoskeletal disorders. Strategies targeting key elements regulating mitochondrial quality exhibit promise in the restoration of mitochondrial function, alleviation of inflammation, and enhancement of overall outcomes.

**Graphical Abstract:**

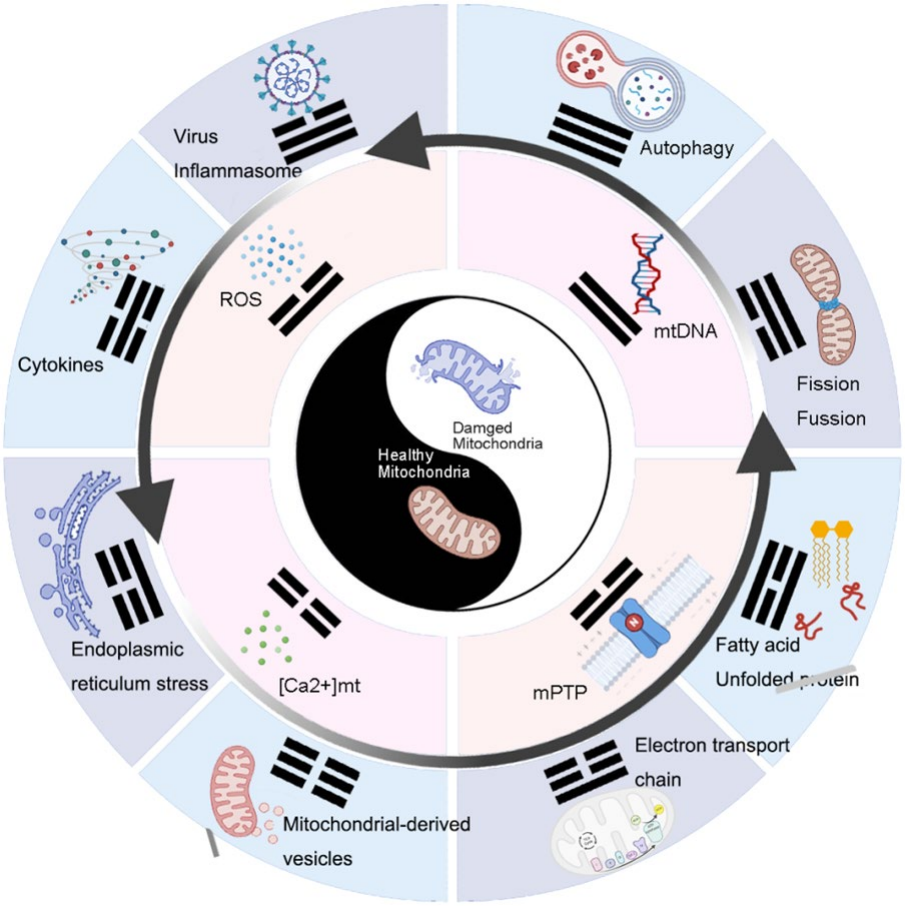

**Supplementary Information:**

The online version contains supplementary material available at 10.1186/s13578-024-01259-9.

## Introduction

Chronic inflammatory musculoskeletal disorders can lead to pain and functional impairment, imposing a substantial physical and economic burden on individual patients as well as society overall. Contemporary investigations have revealed that striated muscles and tendons subjected to persistent inflammation manifest deviations in both microstructural elements and composition. These anomalies encompass diverse features such as calcifications, fibrocartilaginous transformations, and osseous metaplasia. Simultaneously, there is an accompanying diminution in both material and structural characteristics [1]. Consequently, these alterations contribute to a notable decrease in the load-bearing capacity of the affected tissues [2]. The origin of chronic inflammatory musculoskeletal disorders is intricate, involving a myriad of factors. Inherent mechanisms, including the alterations in microvascular blood supply, natural aging process, trauma, and repetitive overuse, collectively contribute to their etiology [3]. Beyond these intrinsic elements, oxidative stress emerges as a significant biological factor implicated in the initiation and progression of chronic musculoskeletal inflammation [4]. The pathological mechanisms involved are still under exploration. At this stage, more and more scholars are focusing on the microstructure of cells, hoping to make a breakthrough.

**Fig. 1:**
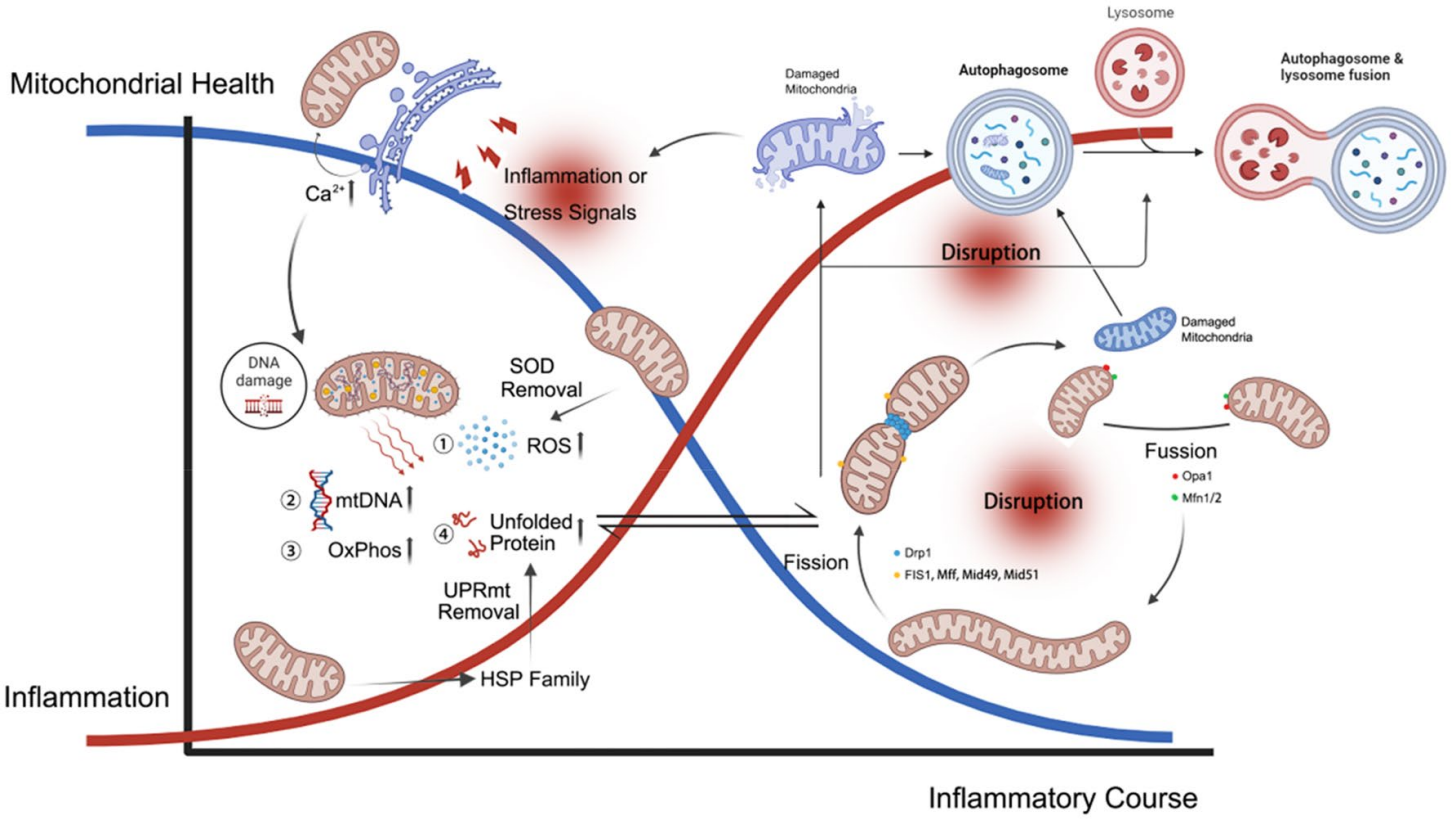
Conceptual model and the corresponding underlying mechanisms that elucidate the relationship between the chronic musculoskeletal inflammation and the mitochondrial damage. The mitochondrial health and inflammatory levels are represented by a blue and red line, respectively. *SOD* superoxide dismutase, *ROS* reactive oxygen species, *mtDNA* mitochondrial DNA, *OxPhos* oxidative phosphorylation, *UPRmt* mitochondrial unfolded protein response, *HSP* heat shock protein. Created with BioRender.com

Skeletal muscle is a biologically active organ that requires a sufficient supply of energy to function properly. Mitochondria, which produce adenosine triphosphate (ATP) through oxidative phosphorylation (OxPhos), are instrumental in providing energy for skeletal muscle [5]. Musculoskeletal diseases, in comparison, can cause mitochondrial defects or abnormalities. Previous research has demonstrated that reactive oxygen species (ROS) bring about oxidative damage in chronic musculoskeletal inflammation, leading to reduced collagen and proteoglycan synthesis, as well as tendon calcification [6]. ROS can trigger and regulate apoptotic processes, making them important factors in chronic inflammatory musculoskeletal disorders. Under normal circumstances, within eukaryotic cells, mitochondria stand as the principal wellspring of ROS. Notably, these ROS predominantly arise from the activities of complexes I and III within the electron transport chain (ETC), a consequence of the reaction between oxygen and electrons escaping from the respiratory chain [7]. In response to the potential onslaught of excessive ROS, mitochondria are equipped with an elaborate antioxidant system [8]. This defense mechanism encompasses enzymes such as superoxide dismutase and glutathione peroxidase, which engage with and effectively eliminate ROS. These mitochondria-located enzymes serve as molecular chaperones, mitigating ROS-induced aberrations in protein folding [9] (Fig. 1). But this antioxidant balance is collectively regulated by various parameters of mitochondria. Relevant studies indicate that factors such as mitochondrial quantity, shape, density, cristae number, and irregularities in tissue organization are all associated with the onset and recovery of chronic inflammatory musculoskeletal disorders [10].

Precisely orchestrated reactive oxygen species (ROS) generation can be instrumental in fostering muscle repair and amplifying muscle contractility. Nonetheless, the systemic or persistent presence of oxidative stress proves deleterious, prompting the activation of mitochondrial quality control (MQC) mechanisms. These include mitochondrial fission and subsequent disposal through autophagy and proteolysis [11]. Consequently, this cascade of events results in a decline in mitochondrial membrane potential, rupture of the outer mitochondrial membrane (OMM), mitochondrial swelling, and substantial structural as well as functional impairments to the mitochondria [12]. A summary of existing literature reveals that factors influencing mitochondrial function encompass defects in mtDNA mutations, mitochondrial OxPhos, structural abnormalities, imbalances in Ca^2+^ levels at the ultra-microscopic level, as well as mitochondrial-associated proteins and chaperone proteins. Additionally, disruptions in the equilibrium between mitochondrial fusion and fission, coupled with compromised lysosomal function stemming from impaired mitophagy and insufficient biosynthesis, collectively contribute to the onset of mitochondrial damage (Fig. 1). Among the above, the most crucial aspects involve three key components: Ca^2+^ imbalance, persistent opening of the mitochondrial permeability transition pore (mPTP), and rampant accumulation of ROS. Firstly, disturbances in mitochondrial Ca^2+^ ([Ca^2+^]mt) homeostasis could instigate Ca^2+^-reliant impairment to mitochondrial structure and performance. The presence of Ca^2+^ in the mitochondrial matrix proves indispensable for the stimulation of oxidative metabolism, as it orchestrates modulation of three rate-constraining enzymes within the tricarboxylic acid cycle (TCA), referred to as pyruvate dehydrogenase, α-ketoglutarate dehydrogenase, and isocitrate dehydrogenase. Under normal circumstances, [Ca^2+^]mt buildup is essential to improve cellular processes and mitochondrial bioenergetics in reaction to cell stressors. During inflammation, as muscle fibers undergo structural fragility, they become notably permeable to the extracellular milieu. This heightened permeability initiates an excessive Ca^2+^ influx, initiating lipases and proteases while imposing an overburden of intramitochondrial Ca^2+^ [13]. Subsequently, an excessively elevated [Ca^2+^]mt could elicit persistent opening of the mPTP [14] (Fig. 1). Two consequential repercussions of mPTP opening are escalated ROS genesis and lowered mitochondrial respiratory capacity.

The interconnected network comprising [Ca^2+^]mt homeostasis, ROS generation, ATP synthesis, and respiration has cascading impacts on cellular function. When molecular irregularities occur within this network, the impact extends beyond the mitochondria, affecting the cytoplasm and nucleus with consequential detrimental effects [15]. First and foremost among these components is mitochondrial DNA (mtDNA), a potent stimulator for innate immune response and inflammation, influencing broader physiological processes. Under normal circumstances, mtDNA is typically enclosed within organelles or encapsulated by membrane structures, shielding it from recognition by the host cell as foreign material. In the context of chronic inflammatory musculoskeletal disorders, such “membrane protective structures” undergo disruption, leading to autoimmune and inflammatory diseases [16]. Moreover, inflammation-induced ATP deficiency prompts a compensatory reliance on cytoplasmic glycolysis. This shift results in elevated lactate levels, reduced pH, and subsequent manifestations of fatigue and compromised excitation–contraction coupling in muscle cells. This interconnected dysfunction extends beyond the molecular level, significantly influencing the overall physiological performance of muscle cells. Deficiencies in mitochondrial number and function hinder fatty acid oxidation, impeding the regenerative capacity of muscle cells. This dual effect underscores the crucial role of mitochondrial health not only in energy metabolism but also in the fundamental processes governing muscle cell regeneration [17]. Thus, maintaining the integrity of this intricate mitochondrial network is essential for overall cellular function and physiological well-being.

How each of the aforementioned factors operates in the development of chronic musculoskeletal diseases, and whether it is due to disruption of the endosymbiotic relationship between mitochondria and cells, remain unknown at present. Reasonable speculations can only be made based on existing research. Simultaneously, the functionality of mitochondria in such inflammation is highly complex, spanning across various hotspots in research. In this review, we aim to systematically and comprehensively summarize and analyze existing literature, with a focus on examining the potential changes in mitochondrial function and the related mechanisms in chronic inflammatory musculoskeletal disorders. Additionally, we will explore the influence of mitochondria on the development and modifications of chronic inflammation.

## Intracellular microenvironment in chronic inflammatory musculoskeletal disorders

It is widely recognized that chronic musculoskeletal inflammation occurs as a result of significant changes in the intracellular environment of skeletal muscle cells. These changes involve various metabolites and inflammatory molecules. The primary factors closely associated with mitochondrial metabolism are unfolded proteins and the buildup of fatty acids. These determinants assume a pivotal role in the initiation of inflammatory processes, exerting a profound impact on both mitochondrial function and cellular metabolism.

### Mitochondrial unfolded protein response

**Fig. 2:**
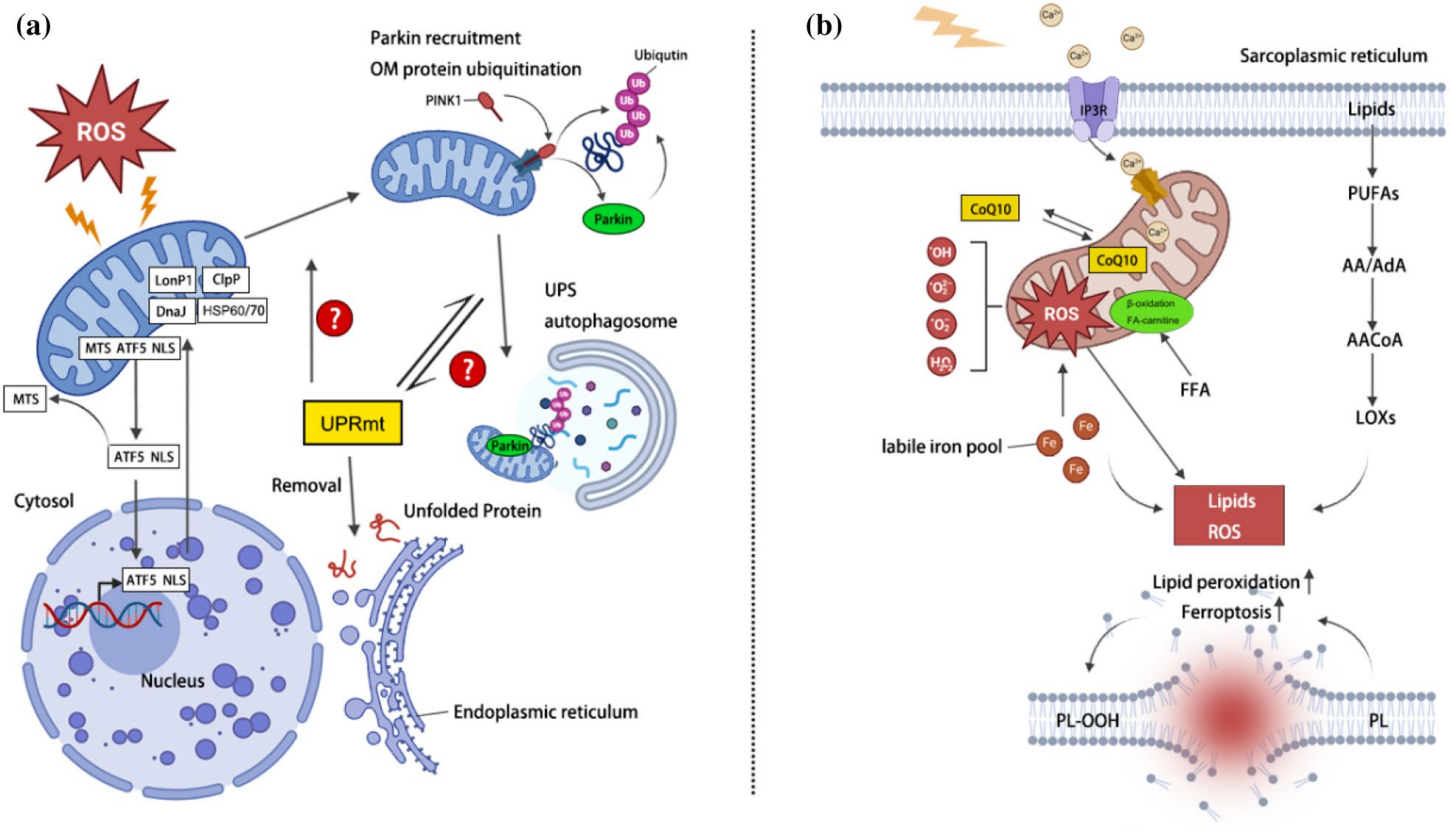
**a** Under stimulation from chronic inflammation, the mechanisms of UPRmt, autophagy–lysosome system, and ubiquitin–proteasome system are activated to clear unfolded proteins. There may be interactions between these systems to coordinate protein quality control processes and maintain cellular homeostasis. Questions regarding how these systems coordinate their operations and the activation thresholds remain unanswered. **b** Regulatory pathways of lipid and ferroptosis. Under the influence of chronic inflammation, abnormalities in fatty acid oxidation and excessive accumulation of oxidized lipids disrupt iron homeostasis and result in the generation of a large quantity of ROS. Fatty acids, unstable iron, and lipids containing PUFAs are key factors in the outbreak of ROS and ferroptosis. The CoQ10 system plays a role in limiting the development of oxidative damage. Eventually, the excessive production of ROS can oxidize phospholipids in cell membranes and lipoproteins into hydrogen peroxide, leading to programmed cell death. *UPRmt* mitochondrial unfolded protein response, *UPS* ubiquitin–proteasome, *PINK1* phosphatase and tensin homolog-induced kinase 1, *HSP* heat shock protein, *ROS* reactive oxygen species, *FFA* free fatty acids, *PUFA* polyunsaturated fatty acids, *AA* arachidonic acid, *AdA* adrenic acid, *LOX* lipoxygenase, *CoA* coenzyme Q10, *IP3R* inositol 1,4,5-trisphosphate receptor, *PL* phospholipids, *PL-OOH* phospholipid hydroperoxides. Created with BioRender.com

Primarily, we have a clear concept that an excess of protein or the improper folding of proteins can have fatal consequences for skeletal muscle cells. Skeletal muscle is not as resilient as cardiomyocytes when faced with protein stress. Therefore, maintaining protein quality control is crucial for skeletal muscle cells [18]. In cases of chronic inflammation in skeletal muscle, unregulated protein aggregation ensues as a consequence of the compromised functionality or excessive demand placed on the autophagy–lysosome systems and ubiquitin–proteasome (UPS). This cascade of events results in the formation of protein aggregates, mitochondrial impairment, the accumulation of hyper-ubiquitinated proteins, and the initiation of autophagy (Fig. 2a). Collectively, these processes ultimately contribute to the induction of muscle fibrotic injury [19]. To effectively manage such situations, mitochondria have developed a highly efficient disposal mechanism, named the mitochondrial unfolded protein response (UPRmt).

The UPRmt is a cellular stress response triggered by unfolded or misfolded proteins within mitochondria, surpassing the capacity of chaperone proteins [20]. Mitochondrial proteins are largely encoded in the nucleus despite having a small genome itself. Therefore, it is evident that mitochondrial function, adaptation, and biogenesis heavily rely on the import of proteins encoded in the nucleus. Notably, the initiation of the UPRmt is prompted by an excess of imported proteins or the occurrence of misfolded proteins. This activation serves as a crucial mechanism to uphold mitochondrial protein homeostasis. It is essential to finely regulate and coordinate these mechanisms to prevent proteotoxicity, which could otherwise lead to dysfunction in both mitochondria and skeletal muscles. Additionally, the UPRmt modulates the activity of genes implicated in OxPhos and the Krebs cycle. This multifaceted response serves to alleviate mitochondrial stress and strategically adjusts cellular metabolism, fostering an environment conducive to cell survival [21].

Numerous proteins and genes participate in the regulation of the UPRmt. Currently, a key focus of research centers around the heat shock protein (HSP) family, recognized for its pivotal part in activating UPRmt. For instance, HSP60 assists in facilitating protein folding within the mitochondrial matrix. It has been identified as a facilitator of enhanced calpain activity, a process integral to protein synthesis, folding, and the transportation of misfolded proteins to proteolytic enzymes within the mitochondrial matrix [22]. Remarkably, the removal of HSP60 triggers a robust proteotoxic stress response, impacting both the mitochondrial matrix and the cytoplasm. This deletion impedes the entry of proteins into the mitochondria. HSP70 is another protein that participates in this process. Under stressful conditions, both tenocytes and myocytes release HSP70, which helps inhibit the activation of NOD-like receptor family pyrin domain containing 3 (NLRP3) inflammasome-mediated pyroptosis and maintain a delicate equilibrium between regenerative and degenerative changes. This observation highlights the potential of HSP70 to act as a link in the loop of death and regenerative [23]. Even with this nearly flawless system, there is still a limit to how many unfolded proteins can be processed before the UPRmt collapses, leading to a significant depletion of HSPs.

**Fig. 3:**
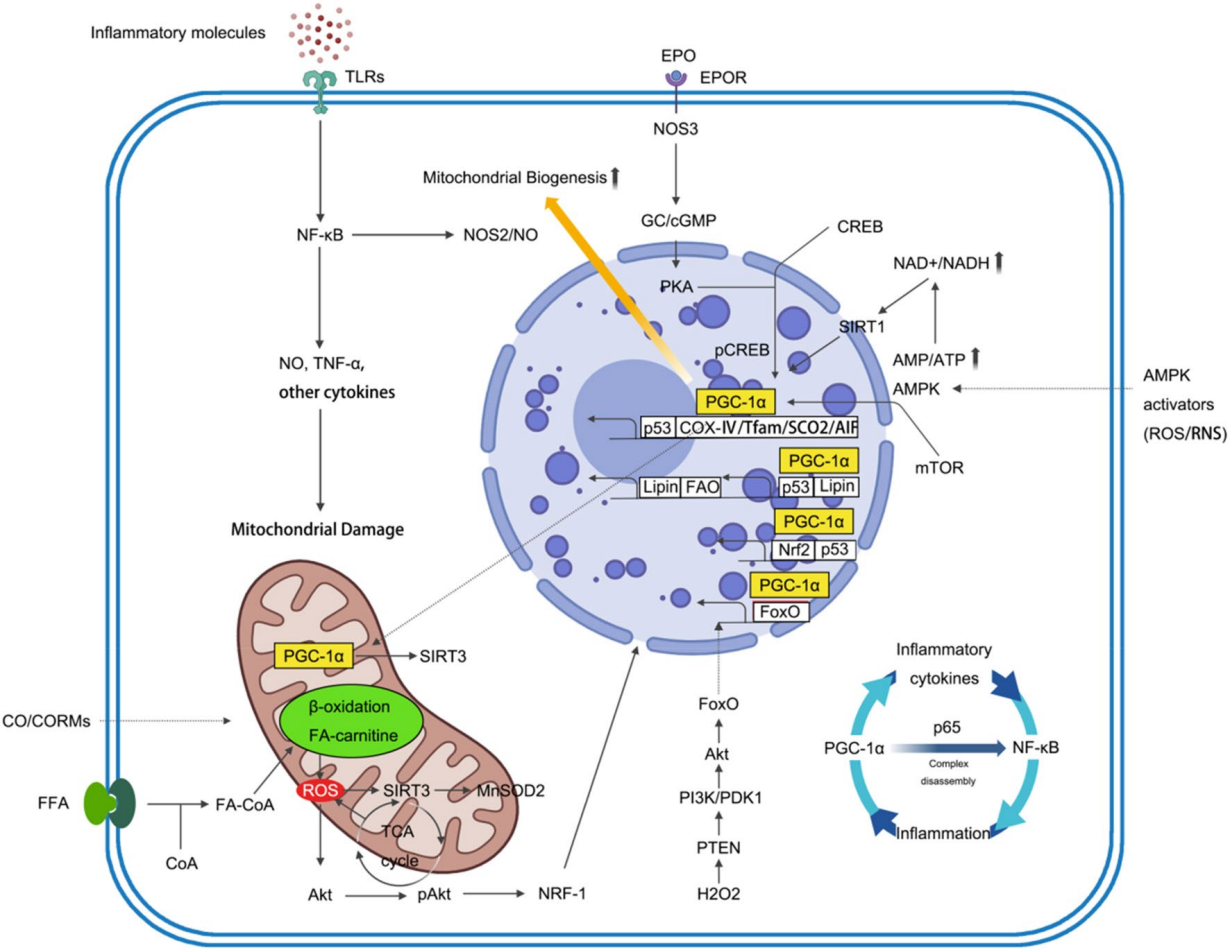
Simplified diagram of a generalized cell illustrating the activation of the PGC-1α signaling pathway and the TLR system on the burst of cytokines, the damage to mitochondria, and the redox signaling pathway for the induction of mitochondrial biogenesis in response to inflammation. *TLR* toll-like receptors, *NF-κB* nuclear factor kappa-light-chain-enhancer of activated B cells, *NOS2* nitric oxide synthase 2, *NO* nitric oxide, *TNF-α* tumor necrosis factor α, *CO* carbon monoxide, *CORM* CO-releasing molecule, *FFA* free fatty acids, *CoA* coenzyme A, *ROS* reactive oxygen species, *RNS* reactive nitrogen species, *TCA* tricarboxylic acid, *MnSOD* manganese superoxide dismutase, *EPO* erythropoietin, *EPOR* erythropoietin receptor, *GC* guanylyl cyclases, *cGMP* cyclic guanosine monophosphate, *PKA* protein kinase A, *CREB* cAMP response element-binding protein, *pCREB* phosphorylated cAMP Response-Element Binding Protein, *NAD* nicotinamide adenine dinucleotide, *AMP* adenosine monophosphate, *AMPK* AMP-activated protein kinase, *mTOR* mechanistic target of rapamycin kinase, *NRF-1* nuclear respiratory factor 1, *PTEN* phosphatase and tensin homologue, *PI3K* phosphoinositide 3-kinase, *PDK1* pyruvate dehydrogenase kinase 1, *FoxO* forkhead box O. Created with BioRender.com

The UPS is the primary protein degradation system within cells, often degrading unfolded proteins prior to UPRmt. The UPS primarily involves two major processes: substrate protein ubiquitination and the subsequent degradation of ubiquitin-tagged proteins by the proteasome [24]. Ubiquitin (Ub) is a 76-amino acid peptide. The process of covalently attaching ubiquitin molecules to substrate proteins, known as protein ubiquitination modification, a cascade catalyzed by ubiquitin-activating enzyme E1, ubiquitin-conjugating enzyme E2, and ubiquitin-protein ligase E3. Proteins marked with ubiquitin can be recognized by the 26S proteasome, or degraded through the autophagy–lysosome pathway, or have the ubiquitin tag removed by deubiquitinase (DUB) catalysis, thereby regulating downstream signaling pathways [24]. Two ubiquitin E3 ligases, Atrogin-1 and muscle-specific RING finger protein 1 (MURF1), are well-known components of this system. Atrogin-1 primarily targets the eukaryotic translation initiation factor 3 subunit F (EIF3F), while MURF1 targets the myosin chain. In muscle cells, the expression of these ligases is modulated by the forkhead box O (FoxO) transcription factor [25]. When muscle atrophy occurs, decreased activation of the protein kinase B family (AKT) promotes the phosphorylation and translocation of FoxO to the nucleus. This, in turn, increases the expression of Atrogin-1 and MURF1, instigating the process of proteolysis [26] (Fig. 3). An additional investigation highlights an augmentation in mitosis during both muscle atrophy and the aging process, coinciding with a decrement in lysosomal function [27]. This suggests that impairment of lysosomal activity may contribute to the accrual of impaired mitochondria. As for how the ubiquitin–proteasome system and UPRmt collaborate, we currently lack specific research evidence (Fig. 2a). However, as chronic musculoskeletal diseases progress, enhancing the effects of both systems in controlling protein substrates might provide a means to inhibit the progression of inflammation.

### Fatty acid accumulation and muscle insulin resistance

The process of fatty infiltration has been observed in both muscle and tendon tissues in instances of chronic inflammatory musculoskeletal disorders. The mechanisms underlying the generation of this fatty infiltration are currently a matter of diverse opinions, lacking a unified perspective. Some speculations can be derived from certain research findings. Firstly, certain proteins, such as peroxisome proliferator-activated receptor γ and fatty acid–binding protein 4, show a significant decrease in tendinopathic tendons compared to intact tendons [28]. Secondly, in individuals with Achilles tendinopathy, a decrease in the expression of markers associated with lipolysis and adiponectin (ADIPOQ) has been noted, coupled with an elevation in markers related to fatty acid β-oxidation [29]. From the above-mentioned studies, it is evident that this fatty infiltration is primarily attributed to the inhibition of native tissue protein synthesis, a reduction in fatty acid metabolism, and an increase in lipid synthesis.

Turning our attention back to skeletal muscle cells, when there is a persistent accumulation of fatty acids, cells need to regulate mitochondria for their breakdown in a process known as fatty acid β-oxidation [30]. According to our knowledge, there are numerous factors that can interfere with this process. The breakdown of fatty acids is hindered when there is a decay in mitochondrial membrane potential, increased mitochondrial mass, abnormal mitochondrial structure, and reduced oxygen consumption [31]. Aberrant fatty acid oxidation is frequently concomitant with the substantial generation of various oxidative stress products, as illustrated in Fig. 3. Particularly, prolonged elevation of inflammatory metabolic byproducts, such as lipid peroxides, can trigger a form of iron-dependent cell death known as ferroptosis [32] (Fig. 2b). Additionally, it stimulates ATP production in mitochondria and induces an elevation in mitochondrial ROS. This, in turn, triggers mitochondrial fission and a decrease in fatty acid oxidation. Persisting accumulation of lipid substances gives rise to another issue, namely, insulin resistance [33]. In myocytes, insulin resistance can cause impaired insulin signaling, thereby disrupting the entry of glucose into skeletal muscle cells [34]. Consequently, these cells undergo degeneration and death under the dual influence of bioenergetic changes and oxidative stress, exacerbating the spread of inflammation.

A positive correlation has been identified between the irregularities in fatty acid oxidation and the explosive generation of ROS. Other studies using models of insulin resistance have indicated that increases in muscular ROS are produced by sources other than the mitochondria. This is due to the buildup of fatty acids and occurs before mitochondrial dysfunction. As a result, there is an increase in mtROS production and fragmentation of the mitochondria, while their biogenesis decreases [35]. While the preceding study offers a somewhat oversimplified explanation of the origins of mitochondrial ROS, it is indisputable that these results uniformly validate the role of lipid metabolism in mitochondrial impairment. This involvement may occur very early and constitute a pivotal factor contributing to the progression and refractory nature of chronic inflammatory musculoskeletal disorders.

## Positional and structural subtypes of mitochondria in chronic inflammatory musculoskeletal disorders

**Fig. 4:**
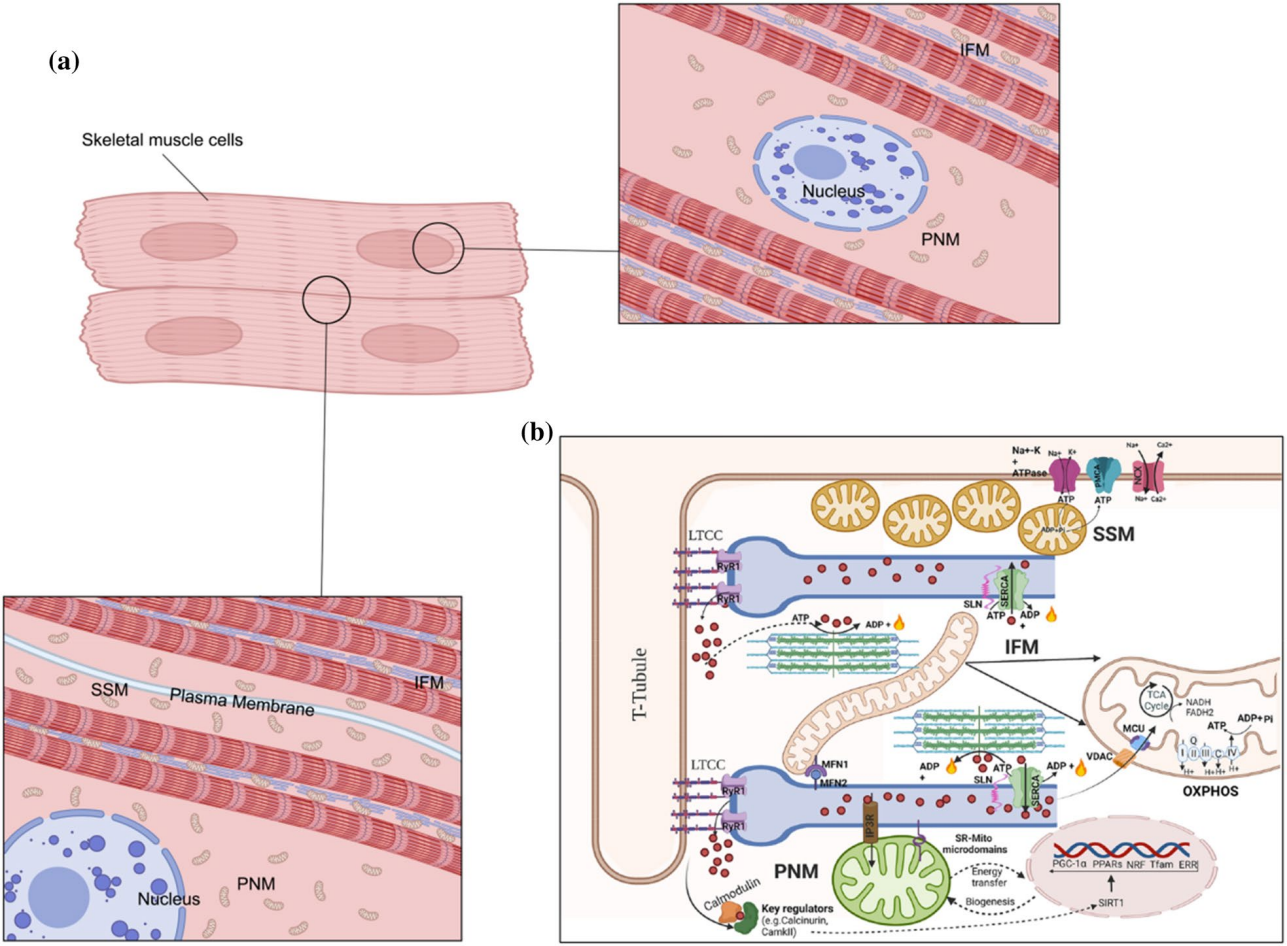
**a** Distribution of different mitochondrial subpopulations in skeletal muscles. In myocyte, the nucleus is clearly surrounded by PNM and SSM subpopulations. Myogenic fibers are interspersed with IFM. The IFM is in close physical proximity to the SR, with numerous contact points. **b** The biogenesis of all subpopulations requires the active participation of the nucleus and the production of chromosome-encoded mitochondrial proteins. While the biogenesis of the PNM and SSM is evident, the IFM appears to have a network-like structure that is well-suited for dynamic activity. The PNM primarily fulfills the energy (ATP) requirements of the nucleus and activates signaling pathways, which in turn activate nuclear transcription factors. The IFM is the major subpopulation responsible for providing energy for muscle contraction, while the SSM supplies energy to the Na^+^–K^+^-ATPase positioned on the sarcolemma. The SR is closely related to the IFM in terms of structure and function, with Mfn1 and Mfn2 playing critical roles in the Ca^2+^ cross-talk between these two organelles. The PNM is intricately linked to the SR in a preferential manner. *SR* sarcoplasmic reticulum, *SSM* subsarcolemmal mitochondria, *PNM* perinuclear mitochondria, *IFM* intra-myofibrillar mitochondria, *ATP* adenosine triphosphate, *ADP* adenosine diphosphate, *OxPhos* oxidative phosphorylation, *LTCC* L-type calcium channel, *RyR* ryanodine receptor, *SERCA* sarcoplasmic reticulum Ca^2+^ ATPase, *SLN* sarcolipin, *MCU* mitochondrial Ca^2+^ uniporter, *VDAC* voltage-dependent anion channel, *TCA* tricarboxylic acid, *NADH* nicotinamide adenine dinucleotide, *FADH* flavin adenine dinucleotide. **a** Was created with BioRender.com. Copyright of **b** obtained from Portland Press and adapted from Swalsingh et al. [246]

In recent years, as we have delved deeper into the study on how mitochondria are distributed and structured, we have been amazed by the intricate network of their functioning. These organelles are arranged in a well-organized manner within cells and carry out a variety of complex tasks through spatial or functional interactions with other nearby organelles or mitochondria. They continuously adapt their morphology, location, and internal structures in response to the external environment. In terms of their subcellular localization, skeletal muscle mitochondria are distinguished into two main categories: intra-myofibrillar mitochondria (IFM) and peripheral mitochondria (PM) [36]. PM can be further classified into subsarcolemmal mitochondria (SSM) and perinuclear mitochondria (PNM) (Fig. 4a).

The IFMs, arranged in rows between myofibrils and filling the space between Z-lines, possess inner membrane characteristics that vary significantly based on the energy levels of the associated myofibrils [37, 38]. This variability in inner membrane features is particularly noticeable in healthy conditions, where IFMs typically display curved cristae structures. In instances of muscle dystrophy and diabetes, the inner membrane of IFMs becomes notably distorted [39]. In contrast, SSM, characterized by variable length and size, can aggregate conspicuously in large numbers under specific circumstances [37]. Adding another layer of complexity, SSM exhibits variations in cristae structure, a feature intricately linked to its metabolic status [40]. On the inner mitochondrial membrane (IMM) of these mitochondria, oxidative complexes are strategically positioned, and the arrangement of cristae facilitates a larger membrane area, contributing to heightened ATP production [41]. The density of mitochondrial cristae is a nuanced factor, often associated with localized energy demand and the overall health of the mitochondria [42]. This sophisticated orchestration within skeletal muscle mitochondria provides valuable insights into the dynamic relationship between structure, function, and the intricate interplay of metabolic demands in maintaining cellular health. Chronic musculoskeletal inflammation can provoke changes in the morphology and density of mitochondrial cristae as well. Research on the specific mechanisms of these changes is lacking empirical support and requires further exploration.

The current body of literature posits that SSM may establish physical connections with the IFM network, potentially optimizing energy distribution within the myocyte. There is evidence suggesting that individual mitochondria from the PM pool can seamlessly merge with the IFM complex, depending on the dynamic metabolic demands of the myocyte [43]. This interchangeability between the two subpopulations underscores a sophisticated regulatory mechanism to adapt to varying cellular energy requirements. The morphological characteristics, including cristae density, are predominantly governed by a delicate balance between mitophagy and dynamics, encompassing the rate of fission and fusion. This equilibrium is intricately linked to the energy needs of the muscle, forming a crucial aspect of cellular homeostasis [44]. Figure 4b illustrates the interconnected relationship between these processes, shedding light on the intricate regulatory mechanisms within skeletal muscle mitochondria. Remarkably, SSM exhibits a heightened responsiveness to outer cues, displaying an increased susceptibility to mitophagy under conditions of chronic inflammation, such as sedentary lifestyles and metabolic diseases [45].

Metabolically, IFM and SSM showcase distinct metabolic capacities dictated by localized intracellular energy demands [37]. Emerging research underscores a tendency for SSM to be more susceptible to dysfunction within the spectrum of mitochondrial sub-populations [44]. The intricate mechanisms governing the configuration of the mitochondrial network, particularly the interplay between IFM and SSM in skeletal muscle, remain elusive. Nevertheless, understanding the overall dynamics of their structural morphology proves crucial for maintaining the hypocellular framework, a foundational aspect of muscle health. Beyond structural nuances, SSM and IFM exhibit variations in degradation rates, enzymatic components, and vulnerability to apoptotic stimulation [46]. These differences highlight the potential for each subpopulation to play unique roles in maintaining cellular homeostasis. Such distinctive contributions may, in turn, have implications for the development of diseases linked to muscle metabolism. The enzymatic machinery governing substrate metabolism (such as lipid or glucose) undergoes substantial modifications during various pathophysiological states [44]. This underscores the dynamic nature of mitochondrial function and its pivotal role in responding to the evolving metabolic demands associated with disease progression. Although we currently lack evidence for this, there is no doubt that this network configuration mechanism could exert a major regulatory role in chronic inflammatory musculoskeletal disorders. We might even speculate that the disruption of this mechanism leads to uncontrolled inflammation.

## Mitochondrial biogenesis in chronic inflammatory musculoskeletal disorders

Maintaining mitochondrial homeostasis hinges on a delicate equilibrium between opposing processes—mitochondrial biogenesis and mitophagy—a dynamic interplay critical for cellular vitality [47]. At the epicenter of this intricate ballet stands peroxisome proliferator-activated receptor-gamma coactivator-1 alpha (PGC-1α), the master regulatory factor orchestrating mitochondrial biogenesis. Serving as a nexus for multiple signaling pathways, PGC-1α activates a cascade of events pivotal for mitochondrial growth and function.

Upon activation, PGC-1α collaborates with essential nuclear transcription cofactors, notably nuclear respiratory factor-1 (Nrf-1) and -2 (Nrf-2), as well as estrogen-related receptor alpha (ERRα) [48]. This collaborative effort culminates in the augmentation of nuclear-encoded mitochondrial proteins (NEMPs) and mitochondrial transcription factor A (TFAM) expression. The intricate orchestration of TFAM includes its translocation into mitochondria, where it propels the expression of mitochondrial genes, forming a robust foundation for biogenesis [49].

The activation of PGC-1α, the linchpin of this process, is finely regulated by two vital players: sirtuin 1 (SIRT1) and AMP-activated protein kinase (AMPK). As both energy sensors and nicotinamide adenine dinucleotide (NAD)+-dependent deacetylases, SIRT1 and AMPK weave together with PGC-1α in a finely tuned regulatory network. This network, sensitive to cellular energy status within the context of energy availability, serves as a sentinel for orchestrating mitochondrial biogenesis and ensuring cellular adaptability [50] (Fig. 3). In essence, the dance of PGC-1α, guided by the harmonious interplay of Nrf-1, Nrf-2, ERRα, TFAM, SIRT1, and AMPK, unfolds as a symphony that intricately balances the scales of mitochondrial biogenesis, ensuring the vitality and adaptability of the cell.

Even the damage caused by the core product ROS in skeletal muscle is primarily counteracted by PGC-1α. In other words, PGC-1α is a central coordinator among exercise benefits, mitochondrial biogenesis, and ROS metabolism. As for how PGC-1α counteracts ROS, it is currently believed to increase the production of antioxidant enzymes by regulating the activity of ERRα [51–53]. Additionally, it might enhance the expression of the SIRT3 gene in mitochondria, thereby increasing the expression and activity of superoxide dismutase 2 (SOD2) [54]. The underlying mechanisms are not yet fully understood. Subsequently, it enhances the ability of mitochondria to clear ROS through these pathways. When PGC-1α is absent, the levels of ROS scavengers like SOD1, SOD2, and glutathione peroxidase 1 (GPx1) decrease. Conversely, when PGC-1α is overexpressed in muscle, the levels of mRNA for SOD2 increase [54]. Henceforth, it is imperative for muscle tissues to uphold a baseline of ROS while simultaneously leveraging these molecules as signaling agents. This dual role serves to augment mitochondrial metabolism, fortify the integrity of the mitochondrial network against potential harm, and mitigate the adverse repercussions that may transpire over prolonged periods [55].

Specifically speaking, the intricate interplay of PGC-1α in cellular responses extends its influence into the realm of inflammatory processes, where it emerges as a pivotal orchestrator. Notably, instances of inflammation witness a noteworthy reduction in PGC-1α levels, thereby intensifying the overall inflammatory response [56, 57]. The specific molecular intricacies underlying PGC-1α downregulation during inflammation remain elusive; nevertheless, the unequivocal involvement of the nuclear factor kappa-light-chain-enhancer of activated B cells (NF-κB) signaling pathway in this regulatory cascade is apparent. At a mechanistic level, PGC-1α governs proinflammatory cytokine levels by directly interacting with the p65 NF-κB subunit [58, 59]. This intricate interplay impedes NF-κB’s transcriptional capabilities, including the expression of genes responsible for proinflammatory cytokines. In the context of skeletal muscle, the absence of PGC-1α precipitates elevated expression levels of tumor necrosis factor α (TNF-α), Interleukin 6 (IL-6), and CD68 [60]. Conversely, the presence of PGC-1α functions as a regulatory counterforce, curtailing the surge in proinflammatory cytokine expression triggered by TNF-α, toll-like receptors (TLR) agonists, and saturated free fatty acids in C2C12 muscle cells [61].

Taken together, the intricate orchestration of optimal biogenesis culminates in the generation of robust and healthy mitochondria—a phenomenon prominently evidenced during physical exercise. Conversely, the presence of inflammatory diseases serves as a significant impediment to the biogenetic process, fostering the creation of compromised and unhealthy mitochondria. In instances of metabolic disorders, there is a discernible reduction in both biogenesis and fusion processes, underscoring the pervasive impact on mitochondrial health within this pathological context.

## Mitochondrial dynamics in chronic inflammatory musculoskeletal disorders

Mitochondrial dynamics encompass the dynamic alterations in the size, shape, and distribution of mitochondria, intricately intertwined with fundamental cellular biological and metabolic processes. The perpetual adaptation of mitochondria to their surroundings involves a continuous process of fission and fusion. This dynamic interplay not only includes the fusion and fission of organelles but also entails membrane remodeling, playing a crucial role in the elimination of aging and damaged mitochondria [62]. The delicate equilibrium of mitochondrial dynamics can be perturbed by various factors, including excessive ROS [63], disruptions in Ca^2+^ homeostasis [64], and active participation in cellular processes such as autophagy [65], mitophagy [66], and apoptosis [67]. The orchestration of these dynamic processes reflects the mitochondria’s responsiveness to cellular cues.

A pivotal determinant in the preservation and adaptation of the mitochondrial network lies in achieving a delicate equilibrium between two essential processes: mitochondrial fission and fusion. Mitochondrial fission serves to break apart and isolate dysfunctional segments of the network, paving the way for their selective degradation. On the other hand, mitochondrial fusion enhances network connectivity, fostering the exchange and sharing of mitochondrial components. This collaborative interplay aims to boost mitochondrial efficiency and overall functionality.

### Mitochondrial fusion

The precise coordination of mitochondrial fusion hinges on the intricate regulation of three pivotal proteins: mitofusin 1 (Mfn1), mitofusin 2 (Mfn2), and Optic Atrophy 1 (Opa1). Both Mfn1 and Mfn2, integral members of the dynamin-related family of large guanosine triphosphate hydrolases (GTPases), share a structurally analogous composition and find their residence within the OMM [68]. In contrast, Opa1 is embedded in the inner mitochondrial membrane (IMM) [68]. A standout among the components constituting the Mitochondria-associated membrane (MAM) is Mfn2, renowned for its pivotal role in fine-tuning MAM structure [69]. In the specific context of skeletal muscle, empirical evidence substantiates the active involvement of Mfn2 in mediating interactions between mitochondria, strategically positioning them in proximity to the sarcoplasmic reticulum (SR) [70] (Fig. 1).

Mitochondrial fusion emerges as an indispensable process, playing a vital role in preserving mitochondrial DNA integrity, sustaining mitochondrial respiration, regulating Ca^2+^ signaling, and maintaining mitochondrial membrane potential—collectively pivotal for cellular health and optimal functionality [68]. The targeted elimination of Opa1 in skeletal muscle sets in motion a cascade of deleterious effects, including mitochondrial dysfunction, heightened oxidative stress, endoplasmic reticulum (ER) stress, and inflammation [71]. Notably, Opa1 deficiency triggers the release of Fibroblast Growth Factor 21 (FGF21) from skeletal muscle, initiating shifts in lipid balance, inflammatory responses, and tissue senescence [72]. These findings underscore the indispensable role of Opa1 in upholding mitochondrial homeostasis. The role of Mfn2 in the development of chronic inflammation is more pronounced. In a study on osteoarthritis, researchers found a significant increase in the expression of Mfn2 in chondrocytes. Knocking down Mfn2 with siRNA reversed age-related metabolic changes in chondrocytes. Overexpression of MFN2 exacerbated the progression of inflammation, while knocking out MFN2 improved it [73]. This suggests that moderate expression of Mfn2 is crucial for controlling chronic inflammation. Effective control of Mfn2 expression may thus become a promising research direction for the treatment of chronic musculoskeletal diseases.

### Mitochondrial fission

Mitochondrial fission, orchestrated primarily by Dynamin-related Protein 1 (DRP1), is a dynamic process critical for cellular homeostasis. DRP1 translocates from the cytosol to the OMM and binds with regulatory proteins such as Mitochondrial Fission 1 (FIS1), Mitochondrial Fission Factor (MFF), MID51, and Mitochondrial Dynamics Protein of 49 kDa (MID49) [74]. This binding initiates the formation of DRP1 oligomers, driving mitochondrial fission. This process segregates damaged mitochondria for subsequent mitophagy and facilitates mitochondrial redistribution during cell division, as illustrated in Fig. 1 [68].

The consequences of mitochondrial fission extend to mitochondrial function, impacting membrane potential, respiration, and ATP production [74]. DRP1’s role in skeletal muscle dynamics is evident in studies showing that Drp1 overexpression impairs skeletal muscle growth [75], while Drp1 deletion or insufficient expression leads to a significant 40–50% atrophy [64, 76]. These findings highlight DRP1’s pivotal regulatory role in shaping skeletal muscle development and maintenance.

An excessive prevalence of fission is linked to metabolic dysfunction within skeletal muscle. When DRP1, the protein responsible for mitochondrial fission, is expressed abnormally, it results in the production of hydrogen peroxide (H_2_O_2_) induced by ceramide, and this in turn impairs mitochondrial bioenergetics [77].

The balance between fission and fusion is crucial for maintaining mtDNA, advancing the cell cycle, regulating metabolism, and producing ATP under normal conditions. The rapid changes in mitochondrial shape that occur after depolarization are not caused by fission, but rather by a reorganization of the IMM. Consequently, the mitochondria take on a donut-shaped, toroidal, or circular appearance [78–81]. In the process of toroid formation, the integrity of the OMM remains intact, while the IMM undergoes substantial reorganization, with the assistance of the IMM protease OMA1 [78]. Notably, the formation of toroids is not contingent on actin and, conversely, is hindered by its presence [80, 81]. It is imperative to underscore that damaged mitochondria possess the ability to undergo splitting, contributing to their subsequent removal through the process of mitophagy [82, 83]. This splitting event might not occur immediately following depolarization. Emerging evidence indicates that mitochondrial fission takes place at a later stage, concurrently with the assembly of autophagosomes and, notably, independently of DRP1 in specific mitophagy scenarios [84]. Considering this perspective, along with the mechanism described above, chronic inflammatory diseases may interfere with the splitting process and promote a high rate of mitochondrial splitting during ultra-endurance activities. This would ultimately worsen the stress response of the mitochondria and ER, disrupting the overall system’s balance.

## Mitochondrial autophagy and chronic inflammatory musculoskeletal disorders

The optimal functioning of skeletal muscle relies significantly on the quantity of mitochondria present. This mitochondrial content depends on the balance between creating new mitochondria through mitochondrial biogenesis and getting rid of damaged components through mitophagy [85]. These two processes regulate the quality and quantity of mitochondrial content in the cell, ultimately establishing an equilibrium that determines mitochondrial density.

Apoptosis has been considered a key feature of chronic inflammatory musculoskeletal disorders and is associated with mitochondrial pathways [86]. Autophagy represents a fundamental intracellular degradation mechanism functioning through lysosomes (Fig. 1). Mitochondria are essential in autophagy by serving as a substrate for degradation [87]. The autophagic machinery serves as a specific shield for them, ensuring sustained energy production during inflammation [88]. The interplay between stress response and mitochondria centered around autophagy is two-fold. Mitochondrial autophagy can remove damaged but intact mitochondria. Under normal conditions, autophagy supports cell renewal, repairs damaged cells [89], and actively participates in cellular differentiation and development [90]. The process of myogenesis involves the crucial step of eliminating underdeveloped mitochondria, which can aid in the restructuring of a more mature mitochondrial complex. Beyond contributing to the establishment of a sophisticated mitochondrial structure, this process is instrumental in mitigating stress signaling. Additionally, the selective removal of dysfunctional mitochondria is paramount for preserving the optimal function of skeletal muscle, acting as a preventive measure against cellular death.

The mitophagy pathways for removing dysfunctional mitochondria can be classified into two main forms [91]. (A) In the Parkin-dependent mitophagy pathway, the stabilization of phosphatase and tensin homolog-induced kinase 1 (PINK1) on the OMMs initiates the recruitment of Parkin. This recruitment process involves modifications such as ubiquitination and phosphorylation of Parkin and ubiquitin (Fig. 2a). A variety of OMM proteins, such as Mfn1, Mfn2, voltage-dependent anion channel (VDAC), Tom20, and Miro, are polyubiquitinated by Parkin during mitophagy. Subsequently, these ubiquitinated proteins are identified by various adaptors, triggering the recruitment of an autophagosome and the subsequent degradation of the mitochondrion. Following ubiquitination, multiple adaptors recognize these modified proteins, facilitating the recruitment of an autophagosome for the subsequent degradation of the mitochondrion. (B) In the Parkin-independent mitophagy pathway, specific mitochondrial proteins, including B-cell lymphoma-2 interacting protein 3 (BNIP3), Nix, NDP52, and FUN14 domain-containing 1 (FUNDC1), function as receptors orchestrating the process of selective mitochondrial degradation. These proteins the mobilization of the autophagosome in proximity to the designated cargo. Benson et al. reported an increase in BNIP3 in degenerative rotator cuff tendon [92], implying a potential link between apoptosis triggered by mitochondrial dysfunction and the progression of chronic inflammatory musculoskeletal disorders.

In addition, ROS appears to wield a substantial influence on autophagy [93], particularly in the context of hypoxia-induced mitochondrial autophagy, where the regulatory oversight is attributed to the protein kinase AMP-activated catalytic subunit alpha 2 (PRKAα2) [94]. Under normal conditions, the levels of mitophagy decrease significantly [95]. A decline in mitophagy has the potential to exacerbate the detrimental loop of abnormal oxidative stress, thereby amplifying inflammation-associated tissue damage [96].

It is noteworthy that the deletion of Mfn2 in skeletal muscle not only diminishes the capacity to regulate autophagy but also hampers the activation of mitochondria. The ablation of Mfn1/2 in skeletal muscle precipitates an increase in mtDNA mutations and subsequent tissue atrophy [97]. Specifically, during chronic inflammation, both autophagy and mitophagy decrease, causing a buildup of dysfunctional mitochondria. When casein kinase-2 (CK2) is specifically removed in skeletal muscle, it results in a decrease in PINK import to the IMM. This leads to an increase in autophagosomes associated with mitochondria, which cannot fuse with lysosomes, ultimately blocking mitophagy.

In recent investigations, numerous studies have underscored the pivotal contribution of various proteins, such as Dystrophia myotonica 1 protein kinase A (DMPK-A), to the realm of inflammatory mitochondrial autophagy [98–102]. For instance, DMPK-A, identified within mitochondria, assumes a critical role in maintaining optimal muscle fiber function and differentiation by functioning as an antioxidant and anti-apoptotic agent [103]. Its interaction and accumulation on the OMM induce structural and morphological alterations in mitochondria, ultimately prompting autophagic processes [104]. The levels of mitophagy-associated proteins, including Parkin, PINK1, BNIP3, and microtubule-associated protein 1 light chain 3 beta (MAP1LC3β), exhibit fluctuations in chronic inflammatory musculoskeletal disorders, thereby instigating mitochondrial degradation. While the precise mechanisms remain incompletely elucidated, it is evident that these proteins wield significant influence in orchestrating the progression of chronic inflammatory musculoskeletal disorders. Importantly, regulating their expression can help alleviate mitochondrial stress responses to some extent and inhibit programmed cell death.

## Mitochondrial bioenergetics and oxidative stress in chronic inflammatory musculoskeletal disorders

The primary function of mitochondria is to furnish skeletal muscle cells with the essential energy needed for their metabolic activities. They are responsible for various biosynthetic and catabolic processes. One of their key functions is to produce ATP through cellular respiration and metabolic regulation. In the intricate milieu of each mitochondrion, ATP is synthesized through the amalgamation of fatty acids, sugars, and amino acids with oxygen. The principal processes governing ATP production encompass the OxPhos and citric acid cycle. During OxPhos, electrons undergo a transfer from NADH to oxygen (O2), traversing through OxPhos complexes I to IV. The Krebs cycle, situated in the mitochondrial matrix, constitutes a crucial phase in this energy-producing cascade. Through this cycle, ATP is generated along with, NADH, flavin adenine dinucleotide (FADH_2_), and carbon dioxide (CO2) [105]. An elevated ratio of ATP to adenosine diphosphate (ADP) and NADH to NAD+ fosters an anabolic environment, facilitating the efflux of metabolites and steering metabolism away from oxygen-independent glycolysis toward biosynthesis [106, 107]. In situations where OxPhos is compromised, as observed in chronic inflammatory diseases, the accumulation of NADH ensues, thereby diminishing the pool of available NAD+. This state of reductive stress prompts a metabolic shift towards mitochondrial anabolic pathways, contributing to the propagation and advancement of inflammation [108].

On the other hand, during inflammation, there is an increase in mitochondrial glutamine breakdown, a decrease in the CoA pool, and an increase in succinate generation, leading to the export of succinate into the cytoplasm through dicarboxylate carriers [109]. Once in the cytoplasm, it can affect the activity of α-ketoglutarate-dependent dioxygenases, crucial for activating factors such as hypoxia-inducible factor 1α (HIF-1α) and IL-1β [110]. These mitochondrial metabolites have the potential to alter DNA and histone methylation, serving as potential epigenetic signals. In addition to these metabolic signals, there is a well-recognized metabolic shift known as the Warburg effect [111]. Its fundamental principle involves increasing flux through glycolysis and the pentose phosphate pathway to provide more metabolic intermediates for cell growth. This shift is not necessarily indicative of mitochondrial dysfunction, as most inflammations require mitochondrial activity [111, 112].

In living organisms, various reactions, both spontaneous and catalyzed by enzymes, can produce O^2−^. These reactions encompass various sources, including the mitochondrial ETC, the plasma membrane-associated NADPH oxidase complex (NOX), the cytosolic xanthine oxidase, and the cytochrome P450 monooxygenases, predominantly located in the ER [113]. Despite their potential for inducing harm, ROS such as superoxide (O^2−^) and its derivatives, particularly H_2_O_2_, function as signaling molecules. They play integral roles in diverse biological responses, including cell proliferation, differentiation, and migration [114]. Accumulating evidence suggests that the rapid production of ROS, such as H_2_O_2_ and O^2−^, constitutes a crucial aspect of the defense mechanism against pathogens [115–117]. A certain concentration of ROS must be maintained in healthy skeletal muscle cells as a crucial signaling molecule and regulator between organelles [118]. There is a perspective suggesting that the elevation of mitochondrial ROS can serve as a mechanism to eliminate bacteria and viruses within cells [119]. Besides H_2_O_2_, other mitochondrially-derived molecules like the TCA cycle intermediates succinate and fumarate have emerged as potent inflammatory signaling factors [120, 121]. Succinate stabilizes HIF-1α in tumors and activated macrophages, inducing a pro-inflammatory transcriptional response [121]. It can stimulate dendritic cells via the succinate receptor SUCNR1 [121]. These metabolites can post-translationally modify proteins, expanding their signaling repertoire [122]. Superoxide does not easily pass through cell membranes and tends to accumulate within cells. Therefore, relying on superoxide dismutase (SOD) for scavenging is often necessary. In contrast, H_2_O_2_ stands out as a neutral and more stable counterpart, exhibiting the distinctive ability to freely traverse membranes. This inherent property renders H_2_O_2_ a more versatile and adaptable signaling molecule in cellular communication and signaling cascades [123]. Intriguingly, mitochondria can selectively release various factors like proteins, nucleic acids, lipids, and metabolites into the cytosol, triggering cell death, innate immunity, and inflammation [124]. This regulated breakdown of the ancient endosymbiotic relationship enables mitochondria to act as intracellular signaling hubs, potentially contributing to inflammatory disease pathogenesis when dysregulated [124].

SOD, an enzyme ubiquitously present in various organisms and situated within the mitochondrial matrix, helps convert superoxide into hydrogen peroxide and oxygen. The loss of SOD activity, therefore, can lead to heightened oxidative damage, manifesting as DNA breakage, protein carbonylation, and membrane lipid peroxidation [125, 126]. This suggests that not effectively removing internally generated superoxide can be harmful. Aside from the harmful effects of superoxide itself, it can interact with nitric oxide to form peroxynitrite (ONOO−), which is another toxic substance [127]. Decomposing ONOO− can produce highly reactive species like OH, NO_2_, and CO^3−^ [128]. SOD stands as the exclusive entity capable of averting the formation of ONOO− by effectively neutralizing O^2−^. Inhibiting SOD reduces the ability to neutralize superoxide radicals, which can then cause damage to mitochondrial components and eventually lead to mitochondrial dysfunction. In a murine model investigating supraspinatus tendinopathy, researchers noted a substantial reduction in both SOD gene expression and activity. Intriguingly, upon the alleviation of subacromial impingement, a converse trend emerged, showcasing an evident increase in SOD activity [10]. From the experiments described above, it can be observed that chronic inflammation stimulation leads to a significant decrease in SOD activity. While the influence of inflammation cannot entirely account for the changes in SOD, it is undeniable that if this stimulation persists, SOD activity will not recover, accompanied by irreversible loss of muscle tissue function.

Based on these recent studies, it is suggested that damaged mitochondria could initiate and activate the inflammatory process, which may hinder the resolution of the typical post-injury inflammatory response. This could result in the development of chronic diseases [129]. Additionally, numerous animal and human studies have indicated a decline in mitochondrial content as individuals age, including a reduction in number, density, and size [130], as well as decreased mitochondrial DNA and protein expression [131]. These mitochondrial impairments can lead to a decrease in ATP production [132], mitochondrial respiration [133], and an increase in ROS production [134]. It has been reported that elderly individuals experience a diminished maximal ATP flux in both their gastrocnemius and soleus muscles [135]. This could potentially explain why the elderly are more susceptible to chronic inflammatory musculoskeletal disorders that are challenging to manage.

## Mitochondria-related cytokines and chronic inflammatory musculoskeletal disorders

Mitochondria that are impaired or dysfunctional emit signals known as mitochondrial-derived damage-associated molecular patterns (mito-DAMPs). These signals are recognized in a similar way as bacterial constituents (PAMPs) by the innate immune system. They are involved in the release of cytokines and the recruitment of inflammatory cells after injury [136]. Upon the compromise of skeletal muscle integrity, mitochondria instigate an acute inflammatory response that is essential to the regeneration of myofibrils. But excessive signaling from dysfunctional mitochondria can have a negative impact on muscle outcomes. Notably, instances such as cyclic stretching of human tenocytes elevate the synthesis of inflammatory mediators like prostaglandin E2 (PGE2) and leukotriene B4 (LTB4) [137], crucial contributors to the onset of chronic inflammation [138].The unrestrained release of mito-DAMPs exacerbates pro-inflammatory mechanisms at the injury site, impeding adaptive responses to oxidative stress and hampering the recovery of functional muscle.

Mito-DAMPs, arising from compromised mitochondrial function, significantly amplify a sustained inflammatory milieu marked by anomalous cytokine release at the injury site [6]. Although the specific cytokines exacerbating muscle stress factors are not yet fully elucidated, a comprehensive retrospective analysis of gene expression underscores substantial elevations in key cytokines—IL-6, IL-1β, chemokine C-X-C motif ligand 1 (CXCL1), and monocyte chemoattractant protein 1 (MCP-1)—in response to diverse muscle injuries. This heightened cytokine profile, identified through a melding of advanced scholarly findings, emerges as a crucial regulatory nexus influencing mitochondrial function and playing multifaceted roles in facilitating optimal muscle recovery. When their levels become abnormal, they can disrupt the healing process by exacerbating mitochondrial dysfunction. Another study has demonstrated that introducing intact mitochondria from an external source can protect tenocytes against TNF-α-induced damage and collagenase-induced tendinopathy [139]. Mitochondrial transplantation exerts an inhibitory effect on NF-κB signaling, concurrently diminishing the expression of pro-inflammatory markers (IL-6 and IL-1β) [140]. This further highlights that healthy mitochondria can effectively regulate and counterbalance the release and expression of inflammatory factors, thereby interrupting the spread of chronic inflammation to some extent.

Chronic inflammatory musculoskeletal disorders commonly occur in an area called the “critical zone” where there is not enough blood supply, leading to incomplete healing [141]. For instance, in this hypoxic environment, the hypoxia-inducible factor subunit alpha (HIF1α) is increased [92]. Oxygen deprivation induces mitochondrial dysfunction, inflammation, and alterations in the metabolic profile, primarily attributed to heightened oxidative stress [142]. Hypoxia triggers the activation of the Na+/Ca^2+^/Li+ exchanger (NCLX), resulting in a notable two- to threefold surge in mitochondrial matrix Na+. This elevated sodium concentration engages with IMM phospholipids, instigating a reduction in membrane fluidity and culminating in the generation of superoxide through semiquinone [143]. Hypoxia plays a critical role in tendinopathy by causing inflammation and cell death. Various transcription factors, including NF-κB [144], tumor protein p53 (TP53) [145], and HIF1α [146], intricately govern abnormal oxidative stress, contributing to heightened expression of chemokines, inflammatory cytokines, growth factors, and cellular cycle controllers. Additionally, heightened oxidative stress exerts inhibitory effects on the AKT/mechanistic target of rapamycin kinase (mTOR) signaling pathway, a pivotal controller of the cellular cycle and protein biosynthesis, culminating in muscle atrophy [93] (Fig. 3). Noteworthy is the pivotal role of HIF1α, responsible for oxygen sensing and modulated by oxygen accessibility and alarmins [147]. Hypoxia induces the translocation of HIF1α into the nucleus in isolated macrophages [148], signifying that HIF1α stimulation leads to mitochondrial dysfunction, marked by heightened levels of mitochondrial mass, mitochondrial membrane potential, and ROS. In contrast, the insulin-like growth factor 1 (IGF1)–AKT–mTOR pathway emerges as the principal signaling cascade regulating muscle mass and protein biosynthesis. Activation of this pathway substantiates an increase in muscle mass [149]. Striated muscle actively produces myokines, amplifying responsiveness to insulin, refining glucose metabolism, regulating carbohydrate and lipid metabolic processes, and exerting considerable influence on bioenergetics and inflammatory responses [150].

In addition to the targets and pathways mentioned above, mtDNA can directly trigger the onset of an inflammatory factor storm. A recent proposition by Shadel et al. posits that mtDNAs play a dual role, not only serving as contributors of nucleic acid and OxPhos components but also functioning as vigilant guardians detecting genomic damage stress and other adversities [151]. In chronic inflammatory musculoskeletal disorders, notable mutations have been detected in mtDNA, encompassing point mutations within protein-coding regions and mt-transfer RNA (mt-tRNA) genes, influencing the synthesis of mitochondrial proteins. Furthermore, substantial deletions in mtDNA have been observed [152]. This observation suggests that the diverse mtDNA genomes within individual cells may serve as a reservoir of signaling molecules. The pathogenicity of mtDNA mutations may be linked to a threshold effect and is associated with serum inflammatory markers [153]. Importantly, mtDNA directly activates the NLRP3 inflammasome and leads to IL-1β production [154]. Inflammation induced by IL-1β has been shown to reduce Mfn1 expression while increasing the level of DRP1 is elevated [155]. mtDNA has undergone continuous evolution marked by the accrual of mutations, giving rise to geographically distinctive and prevalent mtDNA polymorphisms referred to as mtDNA haplogroups. These haplogroups exert a significant influence on essential cellular functions, such as ROS production, bioenergetic output, utilization of oxygen, and the regulation of mitochondrial genes [156]. Substantial evidence now supports the idea that the occurrence, onset, and advancement of chronic musculoskeletal inflammation exhibit variations contingent on distinct mtDNA haplogroups prevalent in diverse populations [157, 158]. Besides mtDNA, mito-DAMPs such as mitochondrial *N*-formyl peptides (NFPs) are detected in trauma patients’ blood circulation at high levels, too. These peptides engage with high-affinity formyl peptide receptors (FPRs), eliciting the recruitment of neutrophils [136].

Inflammatory factors have a direct or indirect impact on both satellite and non-satellite muscle cells [159–161]. These cells are influenced by pro-inflammatory signaling, which can contribute to muscle dysfunction. They serve as a buffer against inflammation, to some extent, by reducing its spread [162]. Excessive inflammation can severely hinder the mitochondrial functionality in satellite cells, leading to compromised myogenic mechanisms and diminished muscle specialization [162]. Our understanding of the mechanisms underlying this aspect remains limited. Perhaps future breakthroughs in research within this domain could serve as the key to controlling the spread of inflammatory processes in the musculoskeletal system. Muscle progenitors, alongside auxiliary cells like fibroblasts, fibroblast precursors (FAPs), and pericytes associated with blood vessels, assist in orchestrating the synthesis of the extracellular matrix (ECM). This intricate network provides a foundational scaffold, fostering the renewal of satellite cells and facilitating the differentiation of muscle cells. The production and regulation of this ECM are influenced by cytokines and immune cells.

## Mitochondrial vesicle, associated membrane, and chronic inflammatory musculoskeletal disorders

The mitochondrial membrane structure and associated extracellular vesicles (EVs) are the physical basis for all its biological activities and functions. Through changes in membrane structure, molecular exchanges with other organelles and various signals are transmitted. This signaling is manifested not only in the delivery and manipulation of instructions, but also in coordination and regional division of work. This is particularly evident in the development of chronic inflammatory musculoskeletal disorders.

### Mitochondrial vesicle

Recently, there has been a growing body of evidence suggesting that specific subpopulations of extracellular vesicles (EVs) contain a wide range of mitochondrial contents. These vesicles, known as mitochondrial-derived vesicles (MDVs), are estimated to be around 70–150 nm in size. Observations have revealed that MDVs possess the capacity to transport mitochondrial components, exerting a discernible influence on the cellular metabolic milieu and phenotypic expressions of target cells [163].

**Fig. 5:**
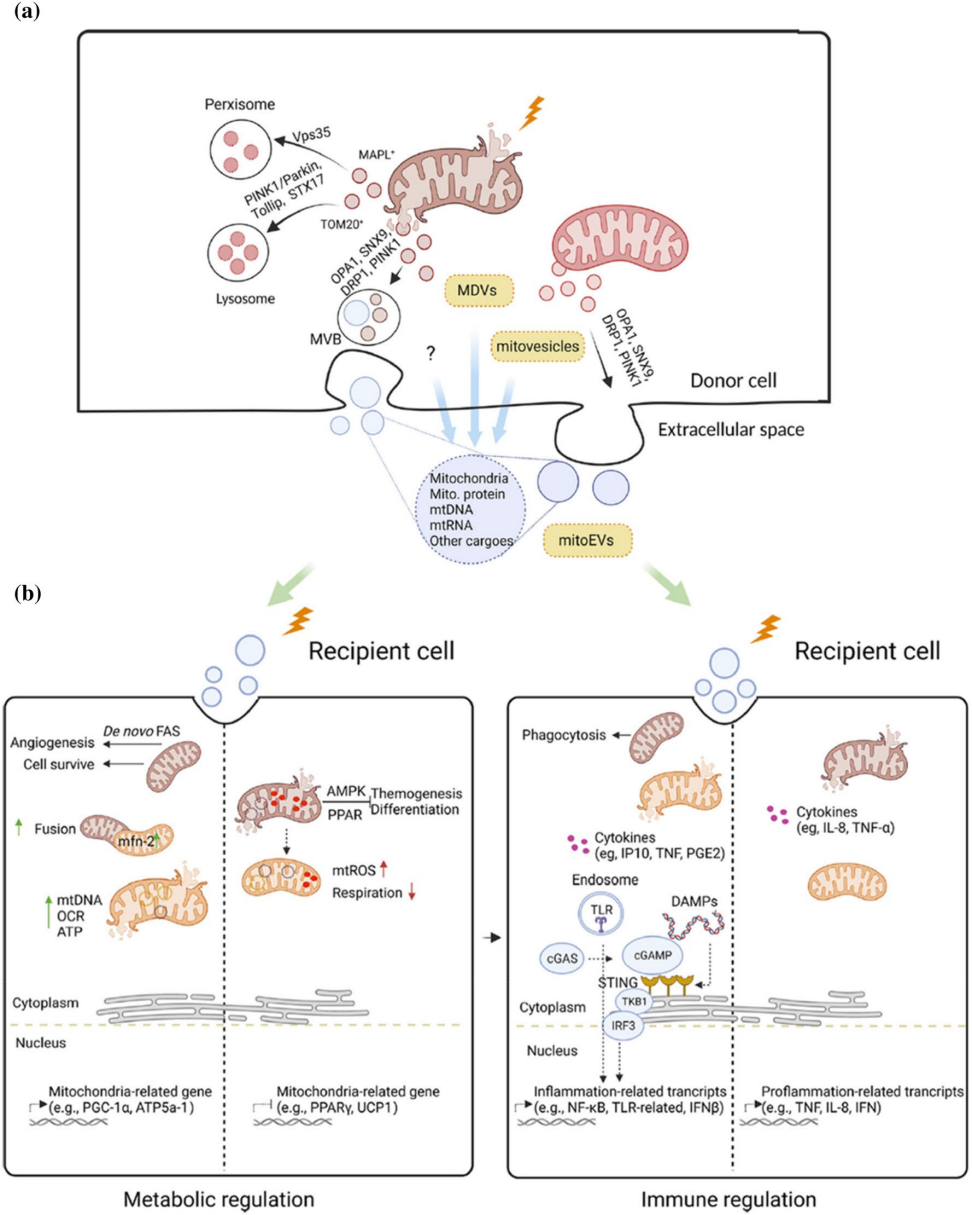
Sorting mechanisms and biological effects of MDVs. **a** Sorting mechanism of donor cells. Damaged tissues under stress release MDVs containing damaged mitochondria, ATP, and mtDNA. These MDVs can be sorted to lysosomes via PINK1/Parkin, Tollip, or STX17, to peroxisomes via Vps35 and MAPL, or to the extracellular space via OPA1, SNX9, DRP1, or PINK1. In some cases, MDVs budding from damaged mitochondria may fuse into MVBs, which are then released into the extracellular space. **b** MDVs carrying oxidized or damaged mitochondrial contents can be taken up by recipient cells or mitochondria through ligand-receptor interactions, endocytosis, or direct plasma membrane fusion. This uptake can affect mitochondrial function and bioenergetics in these cells. The contents of such MDVs can have different downstream regulatory effects, such as mitochondrial biogenesis (e.g., AMPK, PGC-1α), mitochondrial respiration, and bursts of mtROS generation, which mediate cellular phenotypes in recipient cells. MDVs have immunomodulatory roles, including inducing pro-inflammatory signaling in immune cells, cytokine release, IFN response, and phagocytosis in immune cells. *MDVs* mitochondrial-derived vesicles, *ATP* adenosine triphosphate, *mtDNA* mitochondrial DNA, *MVBs* multivesicular endosomes/bodies, *AMPK* AMP-activated protein kinase, *ROS* reactive oxygen species, *IFN* interferon, *TNF-α* tumor necrosis factor α, *OCR* oxygen consumption rate, *PPAR* peroxisome proliferator-activated receptor, *UCP1* uncoupling protein 1, *DAMPs* damage-associated molecular patterns. (Copyright obtained from Wiley Periodicals and adapted from Zhou et al. [247])

MDVs are discharged into the extracellular milieu primarily through two pathways: the multivesicular endosomes/bodies (MVBs) mediated pathway and the micro-vesicle pathway. While the specifics of this process require further clarification, MDVs exhibit the propensity to amalgamate with MVBs and subsequently coalesce with the plasma membrane, potentially implicating OPA1 and SNX9 [164]. These vesicles may undergo liberation through the micro-vesicle pathway. This liberation mechanism can manifest either in a mitophagy-dependent or mitophagy-independent manner, featuring the participation of LC3 and the arrestin domain-containing protein 1 (ARRDC1) as illustrated in Fig. 5a [165]. The liberated EVs are subsequently internalized by recipient cells through a series of intricate processes, including interactions between ligands and receptors, endocytosis, or direct fusion with the plasma membrane. For example, MDVs enriched in damaged mitochondria, when taken up by target cells, exhibit pro-inflammatory properties and stimulate TNF and type I Interferons (IFN) signaling in endothelial cells [166], whereas mitochondria that are phagocytosed by macrophages merge with the existing mitochondrial network and enhance respiration [165].

Correspondingly, under conditions of stress, it is probable that the selective incorporation of mitochondrial contents into MDVs will be promoted, such as oxidative stress [164], remote ischemic preconditioning [167], hypoxia [168], lipopolysaccharide (LPS) [169], and heat stress [169]. Additional investigation is warranted to ascertain the impact of these factors on MDVs released into the extracellular space. The liberation of EVs containing depolarized mitochondria from donor cells experiencing oxidative stress or injury is orchestrated through the ARRDC1 pathway [165]. Notably, myotubes subjected to iron chelation treatment exhibited an amplified sorting of impaired mitochondrial fragments into EVs [170]. This phenomenon persisted irrespective of the inhibition of autophagy or mitophagy [170]. This finding reveals the significance of the ARRDC1 pathway in mediating the release of EVs harboring compromised mitochondrial elements, a process that appears resilient to autophagic and mitophagic interventions. An increase in mitochondrial Lon-induced ROS can trigger mtDNA damage (Fig. 5b). Fascinatingly, the conveyance of mitochondria through EVs has been demonstrated to reinstate mitochondrial function within recipient cells. Specifically, activated platelets exhibit the ability to transmit mitochondria proficient in respiration to mesenchymal stem cells (MSCs), a process attributed, at least in part, to MDVs. This mitochondrial transfer initiates a metabolic reprogramming in MSCs, augmenting TCA activity and de novo synthesis of fatty acids. Consequently, this enhancement contributes to an elevated capacity to promote angiogenesis within MSCs [171]. This explains why platelet rich plasma (PRP) can alleviate chronic inflammation in skeletal muscles and provide therapeutic benefits.

In laboratory conditions, stressed chondrocytes were observed to take up these MDVs and integrate them into their mitochondrial networks. This same phenomenon has been observed in skeletal muscle cells. This discovery strongly substantiates the notion that skeletal muscle cells possess the capability to encapsulate fully functional mitochondria within EVs. Consequently, these mitochondria-laden EVs can be transferred to neighboring cells without necessitating direct cell-to-cell contact [172]. This process is reversed in cases of chronic skeletal muscle inflammation. In such cases, recipient cells can take up EVs harboring oxidized or impaired mitochondrial components, thereby exerting a profound influence on the mitochondrial bioenergetics and functionality within these recipient cells. Tissues undergoing damage due to stress emit EVs enriched with compromised mitochondria, ATP, and mtDNA. Upon uptake of these EVs by neighboring cells, a cascade of events ensues, marked by the activation of the γ receptor, heightened production of mitochondrial proteins, compromised mitochondrial functionality, and the induction of adipogenesis in the recipient cells [173].

The production and content of MDVs within cells have been observed to change in pathological conditions, indicating their potential as biomarkers or targets for treatment in various diseases. Conversely, MDVs originating from healthy cells exhibit the remarkable ability to convey fully functional mitochondrial segments to specific target cells, thus facilitating the restoration of mitochondrial synthesis and energy metabolism [174]. These encouraging discoveries propose that the conveyance of mitochondrial components via EVs holds considerable promise for advancements in disease diagnosis and treatment [175].

Collectively, EVs emerge as pivotal mediators in orchestrating diverse facets of skeletal muscle adaptation and remodeling. Notably, MDVs assume a forefront role in MQC by facilitating the degradation of compromised organelle proteins. Beyond their involvement in the maintenance of mitochondrial integrity, MDVs actively contribute to skeletal muscle’s inflammatory processes and regenerative mechanisms. Their cargo, comprising mtDNA fragments and other essential components, enables MDVs to potentially elicit immune responses, thereby recruiting inflammatory cells and mediators crucial for damaged, aged, and regenerating skeletal muscle (Fig. 5b). In addition, the authors posit that functioning as circulating factors, MDVs might instigate the differentiation of satellite cells in skeletal muscle through the activation of well-established myogenic factors like MyoD and Myf5 [176], exemplifying their multifaceted role in modulating skeletal muscle dynamics [177].

### Mitochondria-associated membrane

Mitochondria, encapsulated by a dual membrane comprising the IMM and OMM, undergo a crucial process termed membrane permeabilization, signifying the release of mitochondrial contents into the cytosol [178]. This intricate phenomenon orchestrates a spectrum of functions, including the transport of Ca^2+^ from the ER to mitochondria, the meticulous regulation of mitochondrial dynamics, the promotion of autophagy, the facilitation of apoptosis signaling, responsive reactions to ER stress, active participation in redox reactions, and the meticulous maintenance of membrane structure.

In skeletal muscle cells, SR stands out as a specialized manifestation of the ER within skeletal muscle, exerting a vital function in an array of cellular processes. These encompass protein biosynthesis, modification, secretion, synthesis of lipids and steroids, and the regulation of Ca^2+^ signaling [179]. Within oxidative muscle fibers, the IFM adopt an intricately organized configuration, forming tightly ordered, elongated structures that establish connections with the SR, extensively branching across the A-band of the sarcomere [180] (Fig. 4a). In both structure and function, these two organelles are intricately linked through the MAM, serving as a dynamic communication platform between them. The MAM actively participates in diverse cellular processes [181], including the conveyance of Ca^2+^ from the SR to the mitochondria, commonly known as SR-mitochondria Ca^2+^ transport. This transport mechanism is facilitated by a molecular complex comprising the inositol 1,4,5-trisphosphate receptor (IP3R), glucose-regulated protein 75 (Grp75), and voltage-dependent anion channel 1 (VDAC1). Remarkably, the intricate network of this complex assumes a critical function in orchestrating the regulation of mitochondrial physiology and maintaining cellular Ca^2+^ homeostasis. Consequently, it emerges as a crucial determinant influencing cellular survival and the delicate balance between life and death [182]. Recent discoveries indicate that the conveyance of Ca^2+^ from the SR to the mitochondria, orchestrated by the macromolecular complex comprising IP3R, Grp75, and VDAC1, may embody a mechanism with universal implications.

According to studies investigating the transport of MAM and SR-mitochondria Ca^2+^ in response to SR stress [183], as well as mitochondrial dynamics [184], speculation abounds regarding the indispensable role of this transport system in facilitating mitochondrial Ca^2+^ overload during intense exercise. Normally, the mitochondrial membrane exhibits direct physical connections to distinct regions of the SR. The transport of Ca^2+^ from the SR to mitochondria is facilitated by the macromolecular complex IP3 R-Grp75-VDAC1, which supplies Ca^2+^ for ATP production. An imbalance in this transport mediated by IP3R-Grp75-VDAC1 can cause diseases and skeletal muscle dysfunction, ultimately contributing to a reduction in muscle performance.

Reportedly, VDAC1 harbors the sites that exhibit an affinity for Ca^2+^, and its closure permits the influx of Ca^2+^ into the mitochondria, ultimately triggering the opening of the mitochondrial permeability transition pore—a critical event associated with programmed cell death. This closure of VDAC1 serves as a signaling mechanism for the initiation of cellular demise. Simultaneously, the mitochondrial Ca^2+^ uptake is influenced by the electrochemical potential difference across the IMM [185]. Notably, two pivotal types of Ca^2+^ release channels reside in the membrane of SR: the ryanodine receptor (RyR) and IP3R, as depicted in Fig. 4b. Upon Ca^2+^ release from the SR, the localized Ca^2+^ concentration in close proximity to the mitochondrial membrane surpasses that in the adjacent cytoplasmic milieu by 5–10 times [186]. During IP3R activation, the concentration of Ca^2+^ at the interface between the SR and mitochondria can further elevate to several tens of micromolar.

Recent research has overturned the aforementioned evidence. The findings of this study suggest that the effectiveness of mitochondrial Ca^2+^ uptake actually relies on the close proximity of mitochondria to Ca^2+^ release channels at the SR membrane. This proximity is crucial for SR-mitochondria Ca^2+^ transport [187]. This discovery implies the direct transportation of Ca^2+^ from the SR to mitochondria, independent of any elevation in cytosolic Ca^2+^ concentration [188]. Regardless of the truth, this mode of Ca^2+^ transport is far more complex than we previously imagined. Functionally speaking, mitochondria function akin to a “Berlin Wall,” acting as a barrier to impede the propagation of cytosolic Ca^2+^ waves originating in the apical area of the cell. On account of this, the cell is compartmentalized into two distinct functional regions marked by discernible cytosolic Ca^2+^ signals [189]. In pathological conditions, a decrease in mitochondrial Ca^2+^ uniporter (MCU) leads to reduced Ca^2+^ accumulation in skeletal muscle mitochondria under-stimulated and resting conditions. Ultimately, this leads to a reduction in muscle fiber size and the onset of muscle atrophy [190]. Examination of the silenced MCU gene highlights a decrease in the respiration rates at both basal and maximal cellular levels, leading to alterations in metabolic processes accompanied by an elevation in the activity of fatty acid pathways [191].

Upon the permeabilization of the OMM by BAX/BAK-associated pores, a cascade of events ensues, culminating in the liberation of critical apoptotic factors such as cytochrome C, apoptotic peptidase-activating factor 1 (APAF1), and somatic (CYTC) from the intramembrane space. This release orchestrates a sequence that ultimately triggers cell apoptosis. Expanding upon this, the permeabilization of the inner mitochondrial membrane (IMM) and the dilation of BAX/BAK pores contribute to the liberation of mtDNA. Therefore, inducing the permeabilization of the OMM can have both a pro-inflammatory effect and activate caspases. The latter process involves the cleavage of pro-IL-1β and pro-IL-18, as well as gasdermin family proteins, ultimately leading to cellular apoptosis [178]. Under physiological circumstances, anti-apoptotic members of the BCL-2 protein family, notably BCL-2 itself and BCL-2-like protein 1 (BCL-2L1), diligently thwart the permeabilization of the OMM. Yet, when confronted with a trigger for apoptosis, either through transcriptional or post-translational mechanisms, BH3-only proteins like BCL-2-binding component 3 (BBC3) and BH3-interacting domain death agonist (BiD) undergo activation. This activation, in turn, leads to the initiation of proapoptotic members within the BCL-2 family, underscoring the dynamic interplay that governs cellular fate in response to apoptotic cues. These proapoptotic proteins oligomerize on the OMM and initiate the permeabilization of the OMM, ultimately causing the release of mtDNA. Whether the released mtDNA is extruded selectively or enters vesicles for further processing remains uncertain.

Subsequent to the release of mtDNA-spillage, the release of factors that induce apoptosis in response to the space between mitochondrial membranes resulting in the activation of an enzyme associated with DNA damage-induced polymerization, specifically poly (ADP-ribose) polymerase (PARP), through a sequence of events mediated by apoptosomes [192]. Abnormal levels of ROS/reactive nitrogen species (RNS), activation of death receptors by their ligands, DNA damage, dysregulation of Ca^2+^, such as cytosolic Ca^2+^ overload, stress in the SR, and alters in the protein production within the BCL-2 family [193].

### Mitochondrial nanotunnels

Inter-mitochondrial junctions (IMJs) are proximal contact sites between OMMs, sharing structural similarities with cell–cell gap junctions. Initially identified in cardiomyocytes connecting electrically coupled mitochondria, these junctions are characterized by highly electron-dense mitochondrial membranes [194]. The frequency of IMJs escalates in tandem with cellular energy demands and mitochondrial volume density [40]. Mitochondrial nanotunnels, thin double-membrane protrusions approximately 100 nm in width, extend from donor mitochondria across distances of several microns. These nanotunnels demonstrate the capacity to interact and fuse with recipient mitochondria [195].

While in the majority of cellular microenvironments, mitochondrial dynamics are well-suited for routine metabolic activities. In differentiated cells with a dense cellular structure environment, like skeletal muscle cells, the movement of mitochondria is considerably restricted. This acts as a robust hindrance, akin to a ship anchor, limiting the trajectory of mitochondrial activity and consequently reducing the frequency of potential mitochondrial fusion events [196, 197]. At this point, the role of mitochondrial nanochannels becomes particularly evident. Strikingly, these dysfunctional mitochondria were observed to possess six times more nanotunnels compared to their counterparts in healthy controls [197]. Intriguingly, mitochondrial nanotunnels may arise or stabilize in a functionally complementing manner, particularly between mitochondria harboring impaired OxPhos capacity [195]. The author posits that mitochondrial nanochannels represent communication structures generated by stationary mitochondria “seeking assistance.” This interpretation draws parallels with bacterial “quorum sensing” behavior [198]. Considering the bacterial origins of mitochondria and their retention of specific functions and structural traits inherited from their prokaryotic ancestors, the existence of tubular protrusions in mitochondrial membranes facilitating molecular exchange does not appear entirely unexpected.

Relevant studies indicate that prolonged disruptions in Ca^2+^ homeostasis can lead to mitochondrial stress, potentially serving as a crucial triggering factor for the formation of nanotunnels [199]. This can be ascribed to the disturbance in Ca^2+^ dynamics, hindering the process of mitochondrial fusion, as Ca^2+^ peaks are essential for maintaining normal fusion events. Such disturbances significantly impact the complementary functions and molecular interactions between mitochondria. 3D reconstruction of the mitochondrial network in human skeletal muscle reveals that both failed fission and re-fusion events give rise to new nanochannels [197, 200]. Further research is needed to confirm whether mitochondrial nanotunnels have an impact on chronic inflammatory musculoskeletal disorders.

## Relationship between migrasome, mitochondria, and chronic inflammatory musculoskeletal disorders

The migrasome is a recently identified organelle that is formed by moving cells. In the course of cell migration, retraction fibers are drawn from the posterior of the cells, leading to the formation of migrasomes—substantial vesicular structures—on these retraction fibers. Similar observations have been made with other organelles within the cell [201–204]. Migrasomes aid in the removal of damaged mitochondria in migrating cells, which helps to preserve MQC [203]. This process is known as mitocytosis [205]. The Drp1 assumes a pivotal role in mitocytosis. Drp1 likely facilitates mitocytosis by promoting the fission of damaged mitochondria from the mitochondrial network—an essential process in preserving the potential across the mitochondrial membrane under conditions of mild mitochondrial stress. This discovery opens up new avenues for further exploration of mitochondria.

Presently, migrasomes exhibit a triad of functions: the release of signaling molecules through rupture or leakage, acting as carriers for damaged mitochondria, and enabling the lateral transfer of mRNA and proteins [206]. This versatility is exemplified in experiments with heteroplasmic cells housing both normal and mutant mtDNA-containing mitochondria. The mutant mtDNA, marked by a significant deletion of genes encoding electron transfer chain proteins, predominates within migrasomes derived from these cells. This observation indicates a preferential transport of functionally impaired mitochondria into migrasomes. The selectivity in this process is attributed to the distinct binding tendencies of damaged mitochondria to motor proteins. Damaged mitochondria exhibit avoidance in binding to the inward motor protein Dynein, while exposure to mitochondrial stressors enhances their affinity for the outward motor Kinesin 1. Consequently, damaged mitochondria are transported to the cell periphery, where they are sequestered into migrasomes and eventually eliminated. At the cell periphery, mitochondria tether to the plasma membrane through the action of Myosin19, an actin-based motor protein renowned for its binding to cortical actin and mitochondria [205].

Mitocytosis is particularly involved in addressing instances of mild mitochondrial damage, typically encountered within the realm of normal physiological conditions [205]. By using in vivo imaging, observations by researchers indicate that neutrophils produce substantial quantities of migrasomes in the circulatory system [205, 206]. These migrasomes often contain mitochondria with abnormal morphology. To further investigate this phenomenon, the researchers conducted a study using Tspan9 knockout mice, which have reduced migrasome formation [207]. It was found that the circulating neutrophils in these mice had significantly decreased mitochondrial membrane potential and viability. These findings imply that the elimination of impaired mitochondria is crucial for maintaining the survival of circulating neutrophils. When cells initiate migration, migrasomes are formed to facilitate mitocytosis, which helps balance out the increased mitochondrial stress caused by the higher energy demand [208]. In this way, mitocytosis integrates MQC with cellular migration. It is crucial to emphasize that even the aggregation of a limited quantity of impaired mitochondria can exert a significant long-term impact [209]. The continuous elimination of impaired mitochondria through mitocytosis empowers cells to evade the adverse consequences linked to the accumulation of dysfunctional mitochondria. Currently, the exploration of migrasomes’ role in disease is at its nascent stage. In order to manage mitochondrial stress, mitocytosis and mitophagy might be employed as a two-step system. Mitocytosis handles mild mitochondrial stress in such a system, while mitophagy handles severe mitochondrial damage. Further investigation is needed to understand how this process operates in the context of chronic inflammation. It is undeniable that there is a significant presence of damaged and mutated mitochondria in chronic inflammation [210], which may be attributed to the activity of migrasomes.

## Relationship between virus, mitochondria, and chronic inflammatory musculoskeletal disorders

Many viral diseases disrupt the functioning of mitochondria [211]. For example, the Epstein–Barr virus (EBV) impacts mitochondrial fission [212], while the pseudorabies virus (PRV) and herpes simplex virus type 1 (HSV-1) alter the balance of Ca^2+^ [213]. In addition, several viruses, such as the Hepatitis B virus and influenza viruses, endorse and produce proteins that promote programmed cell death [214]. Muscle biopsies are infrequently conducted in suspected instances of virus-related myositis, leading to limited data on the underlying pathophysiological mechanisms behind acute or chronic musculoskeletal symptoms during viral infections [215]. Musculoskeletal symptoms or myopathies, occurring not only in the acute phase of viral infections but also in postinfectious syndromes (PIS), are notably linked to overlapping syndromes like chronic fatigue syndromes (CFS), wherein viral infections are believed to contribute to the development of the condition [216, 217].

Following the emergence of unidentified pneumonia cases in Wuhan hospitals, China, in late 2019, and the subsequent categorization by the World Health Organization (WHO) as severe acute respiratory syndrome coronavirus 2 (SARS-CoV-2), the ailment quickly spread globally and became a pandemic within a short span of 3 months. Numerous reports have documented muscular symptoms during the post-acute phase of COVID-19 [218]. In accordance with an extensive longitudinal cohort study, 52% of individuals surveyed 6 months post-infection reported the presence of muscle fatigue and weakness, and this percentage remained at 20% even after 1 year of infection. Women were found to have a higher prevalence of these symptoms compared to men [219]. The study found that 3% of individuals experienced myalgia after 6 months, which increased to 4% after 12 months.

**Fig. 6:**
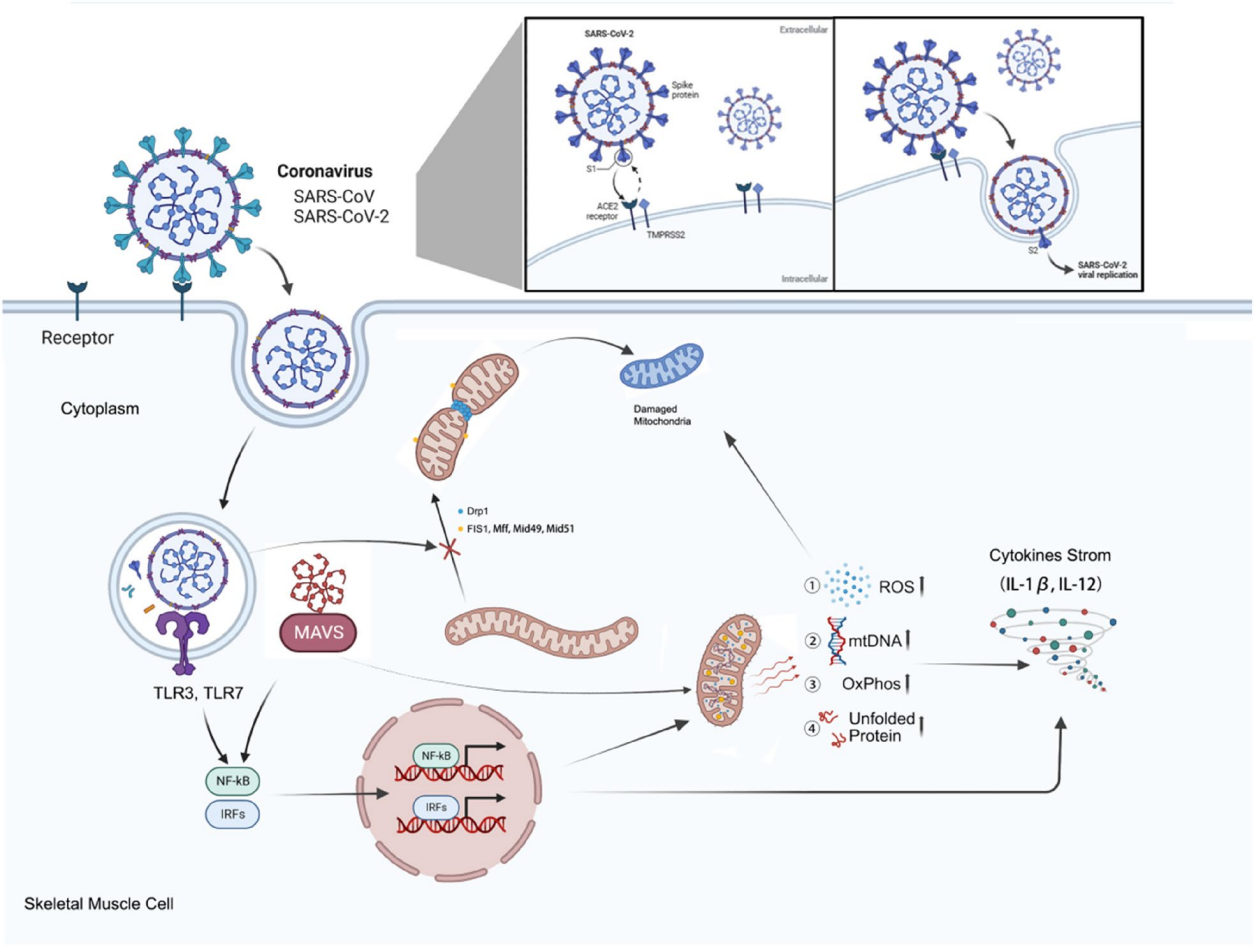
Schematic representation of how COVID-19 causes damage to mitochondria and leads to chronic skeletal muscle disease. SARS-CoV-2 infects skeletal muscle cells directly through the ACE2 receptor. The viral RNA is recognized by the PRR in the cytoplasm, activating the MAVS and type I/III response. SARS-CoV-2 has developed mechanisms to evade the innate immune response, such as inhibiting MAVS through impaired mitotic function. Impaired mitophagy results in mitochondrial dysfunction. SARS-CoV-2 exploits the mitochondrial machinery for its own replication, further contributing to mitochondrial dysfunction. Mitochondrial damage, the release of free mtDNA, and uncontrolled viral replication all enhance inflammasome activation and cytokine storms, ultimately causing mitochondrial death. *ACE2* angiotensin-converting enzyme 2, *MAVS* mitochondrial antiviral signaling, *TLR* toll-like receptors, *mtDNA* mitochondrial DNA, *ROS* reactive oxygen species, *PRRs* pattern recognition receptors, *NF-κB* nuclear factor kappa-light-chain-enhancer of activated B, *IRFs* interferon regulatory factors, *IL* interleukin, *SARS-CoV-2* severe acute respiratory syndrome coronavirus 2. Created with BioRender.com

Since the start of the pandemic, we have been studying the potential immunological mechanisms involved in the development of autoimmune diseases after SARS-CoV-2 infection. Eventually, our focus shifted to the mitochondria. SARS-CoV-2 infection causes stress and damage to the mitochondria. Several studies suggest that the spike protein can harm the mitochondria of human cells. SARS-CoV-2 can directly infect skeletal muscle cells through angiotensin-converting enzyme 2 (ACE2) and activate resident immune cells, resulting in both direct viral damage and indirect immune-mediated damage [220]. The virus attacks the mitochondria, particularly the OxPhos pathway, such as Complex-I [221], which leads to abnormal production of ROS. The heightened generation of ROS and the subsequent release of oxidized mtDNA into the cytoplasm further exacerbate inflammation by activating the NLRP3 inflammasome, initiating the inflammation cascade [222, 223]. Activation of the inflammasome leads to the synthesis of pro-inflammatory cytokines, like IL-1β and IL-12, thereby augmenting susceptibility to chronic inflammation [224] (Fig. 6).

In addition, investigations into metabolism propose that SARS-CoV-2 hinders mitophagy [225], causing the buildup of malfunctioning and compromised mitochondria. This not only impairs the response of the mitochondrial antiviral signaling protein (MAVS), but also worsens inflammation and cell death [226–228]. Nonetheless, the precise molecular mechanisms underlying this process warrant further critical evaluation. The hijacking of mitochondria by SARS-CoV-2 may additionally jeopardize their integrity, impede their functionality, and elevate oxidative stress. SARS-CoV-2 alters the dynamics of mitochondria by affecting processes/functions such as autophagy [229], UPR stress [230], mitophagy [231], and enzymes involved in these processes [232] (Fig. 6). Hence, SARS-CoV-2 has a significant impact on mitochondrial physiology [233, 234]. Based on the aforementioned research, it is conceivable that exogenous supplementation of healthy mitochondria or extracellular vesicles secreted by mitochondria could be utilized for treating chronic musculoskeletal inflammation induced by pathogenic agents such as SARS-CoV-2 and similar pathogens. This may offer novel insights and directions for future research endeavors. It has been suggested that COVID-19 may trigger or worsen idiopathic inflammatory myopathies (IIMs), which are chronic autoimmune-mediated muscle injuries. The discussion of these autoimmune conditions is beyond the scope of this review.

## Future directions

Exploring the intricate mitochondrial mechanisms underlying the pathogenesis of chronic inflammatory musculoskeletal disorders reveals their pivotal role as regulators of regulated cell death (RCD) [235]. The evolving evidence establishes a close association between disruptions in mitochondrial functions and structure during RCD, triggering an inflammatory response essential for maintaining organismal balance [236]. This dysregulation of inflammatory responses, attributed to mitochondrial components, contributes to various human disorders, spanning conditions characterized by excessive inflammation to those arising from inefficient inflammatory reactions [237].

Current clinical interventions predominantly target the effector phase of inflammation, employing therapies like cytokine-neutralizing agents and addressing pattern recognition receptors (PRRs) such as interferon response cGAMP interactor 1 (STING1) agonists [238]. Despite the focus on these strategies, the modulation of inflammation through targeted mitochondrial interventions remains an underexplored avenue. This may stem from the nascent state of research in this domain and the limited pharmacological interventions available for mitigating mitochondrial dysfunction in patients. Currently, the exploration extends to mitochondrial transplantation as a potential therapeutic approach for chronic injury [239, 240]. Studies suggest that MSCs facilitate mitochondrial exchange with damaged cells, offering a potential avenue for tissue recovery [241]. Despite encouraging in vitro evidence, further in vivo experimental research is imperative to validate the efficacy of stem cell-derived mitochondrial transplantation for treating chronic inflammatory injury [242].

Navigating the critical challenge of mitochondrial transfer, compatibility with nuclear DNA (nDNA) emerges as a pivotal factor. Enhanced mtDNA–nDNA pairing and improved mtDNA–nDNA metabolic profile pairing demonstrate favorable effects on cell proliferation [243, 244]. The “Mitopunch” tool expands genomic combinations, addressing the complexities of mtDNA–nDNA compatibility [244]. Beyond nDNA interactions, establishing robust functional connections between exogenous mitochondria and other cellular organelles is crucial for cellular homeostasis and preventing disease [245]. Yet, the intricacies of mitochondrial fusion with endogenous counterparts, alterations in recipient cell or mitochondrial autophagy, and selective autophagy of exogenous mitochondria pose important research directions for further investigation. The ultimate objective is to develop strategies targeting mitochondrial functions to finely control inflammatory reactions in patients.

### Supplementary Information

Supplementary Material 1.

## Data Availability

Data sharing is not applicable to this article as no datasets were generated or analysed during the current study.
