# Supplementary material for: Mitochondrial mechanisms in the pathogenesis of chronic inflammatory musculoskeletal disorders
Source: Cell Biosci. 2024 Jun 8;14:76. doi: 10.1186/s13578-024-01259-9 (PMC11162051; doi:10.1186/s13578-024-01259-9)
Supplement: Supplementary file 1 — Supplementary Material 1. [file 13578_2024_1259_MOESM1_ESM.pptx]

## Slide 1
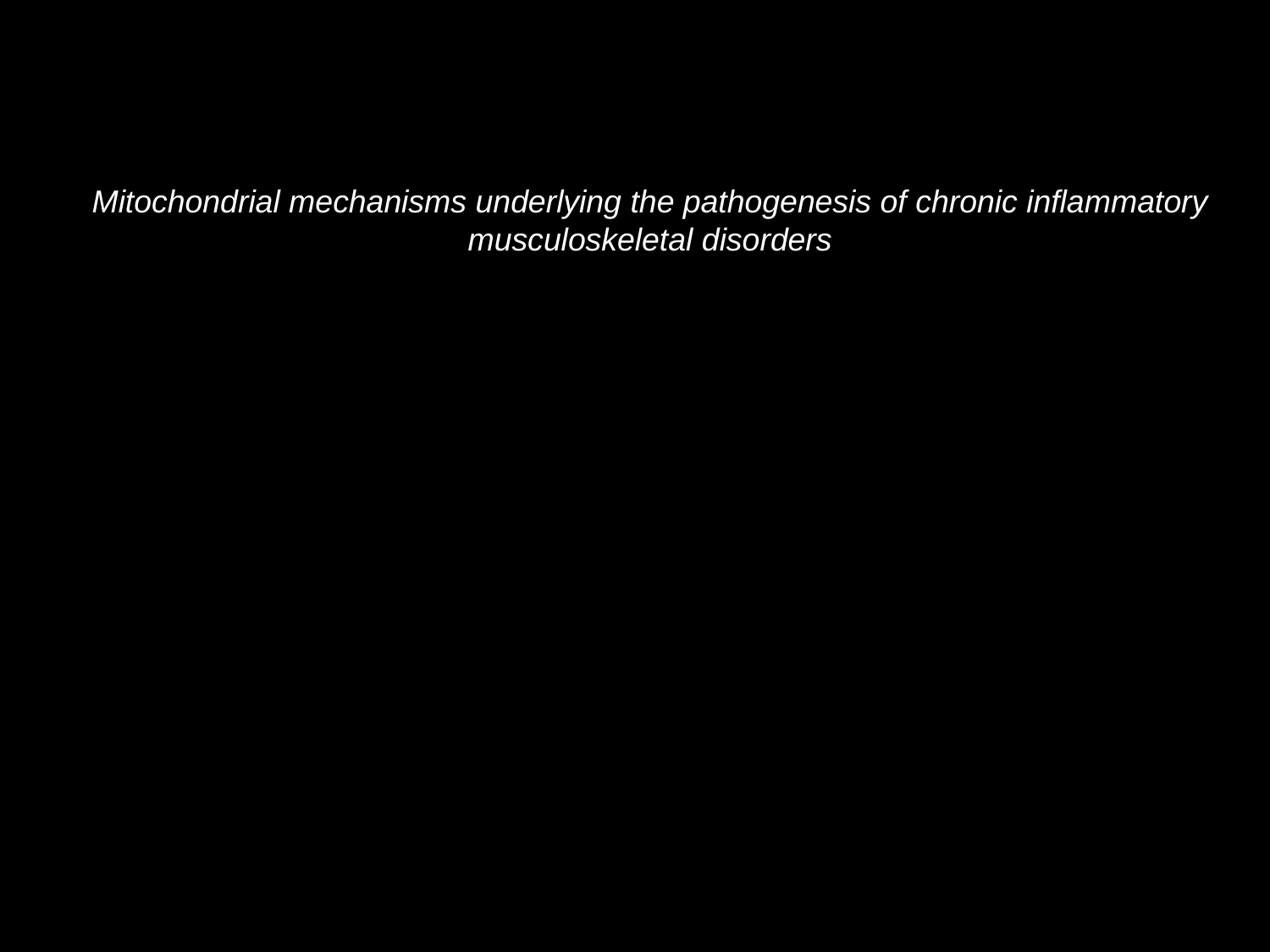

Mitochondrial mechanisms underlying the pathogenesis of chronic inflammatory musculoskeletal disorders

## Slide 2
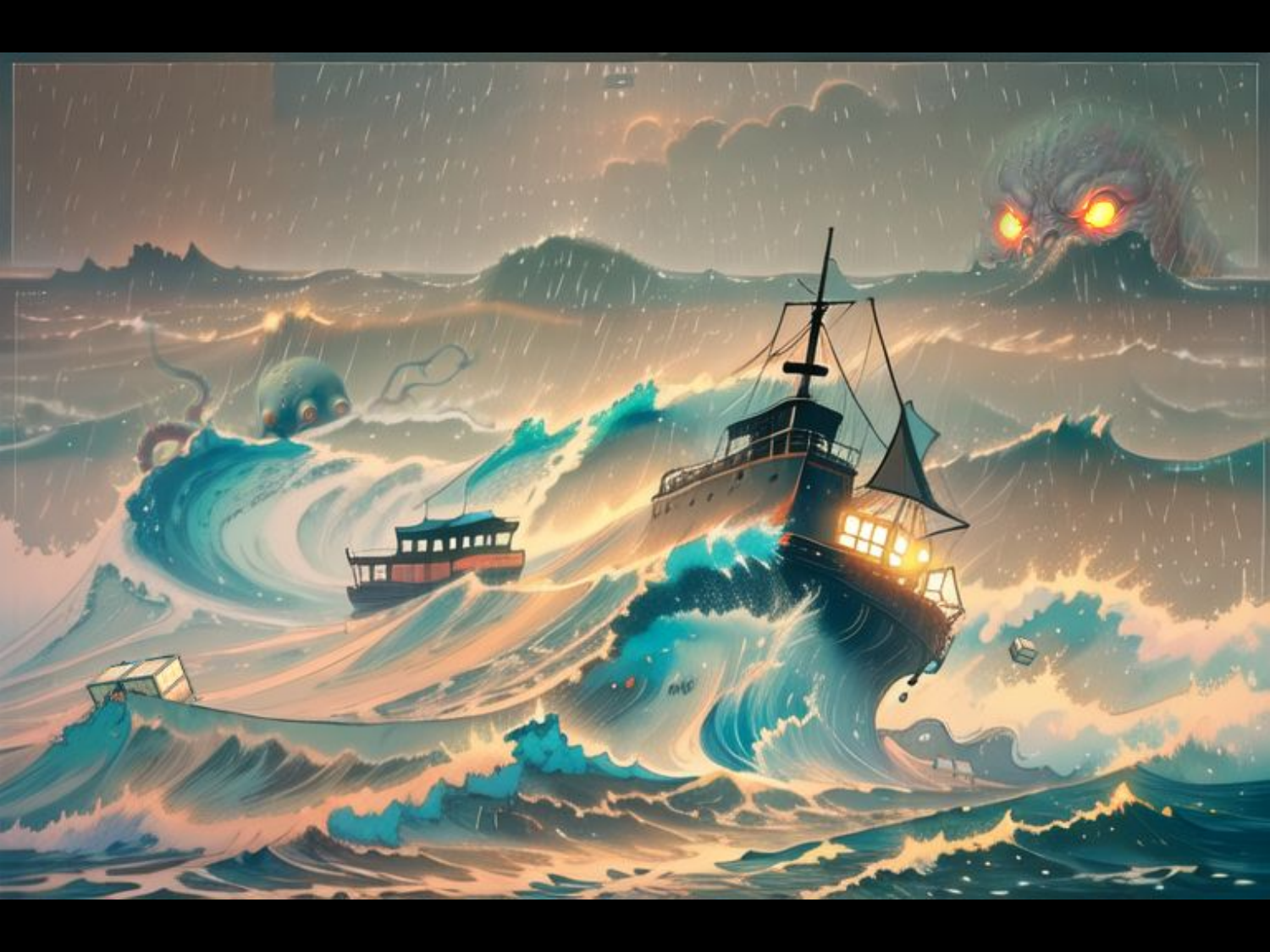

## Slide 3
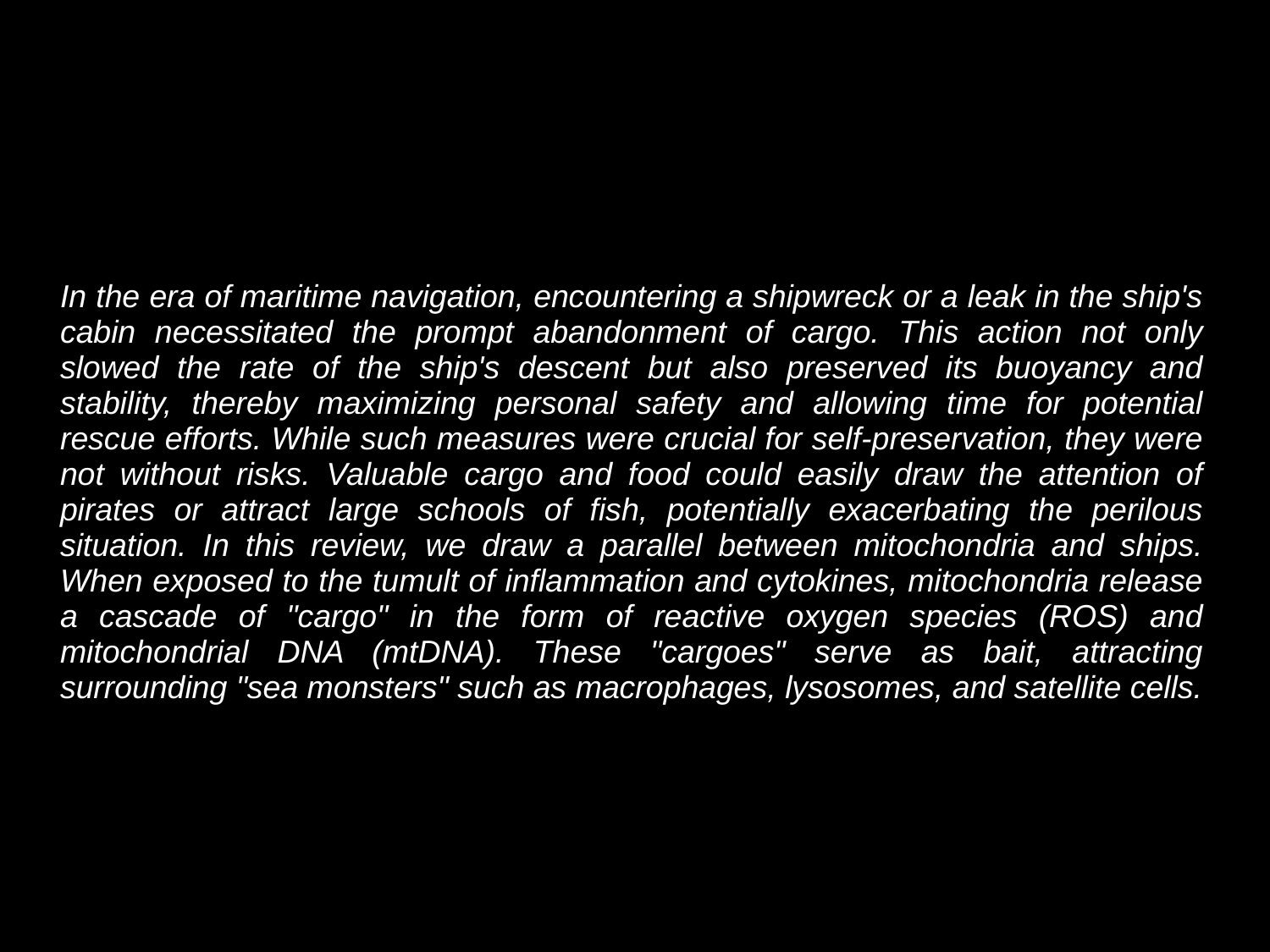

#
In the era of maritime navigation, encountering a shipwreck or a leak in the ship's cabin necessitated the prompt abandonment of cargo. This action not only slowed the rate of the ship's descent but also preserved its buoyancy and stability, thereby maximizing personal safety and allowing time for potential rescue efforts. While such measures were crucial for self-preservation, they were not without risks. Valuable cargo and food could easily draw the attention of pirates or attract large schools of fish, potentially exacerbating the perilous situation. In this review, we draw a parallel between mitochondria and ships. When exposed to the tumult of inflammation and cytokines, mitochondria release a cascade of "cargo" in the form of reactive oxygen species (ROS) and mitochondrial DNA (mtDNA). These "cargoes" serve as bait, attracting surrounding "sea monsters" such as macrophages, lysosomes, and satellite cells.
